# Shape‐Selective Molecular Separations Enabled by Rigid and Interconnected Confinements Engineered in Conjugated Microporous Polymer Membranes

**DOI:** 10.1002/advs.202416266

**Published:** 2025-04-17

**Authors:** Yanqiu Lu, Hao Deng, Liling Zhang, Yong Wang, Sui Zhang

**Affiliations:** ^1^ Department of Chemical and Biomolecular Engineering National University of Singapore 4 Engineering Drive 4 Singapore 117585 Singapore; ^2^ School of Energy and Environment Southeast University No. 2 Sipailou Nanjing 210096 P. R. China; ^3^ Cambridge Centre for Advanced Research in Energy Efficiency in Singapore 1 Create Way Singapore 138602 Singapore; ^4^ Institute of High Performance Computing (IHPC) Agency for Science Technology and Research (A*STAR) 1 Fusionopolis Way, #16‐16 Connexis Singapore 138632 Singapore

**Keywords:** conjugated microporous polymer, shape‐selective molecular separation, small organic mixture separation, solvent‐resistant membrane

## Abstract

Separating molecules with similar sizes but different shapes is essential yet challenging. Here, conjugated microporous polymer (CMP) membranes with narrowly distributed network pores are prepared by diffusion‐modulated electropolymerization. This approach precisely controls the monomer diffusion and coupling processes, regulating the crosslinking degree to prevent broad aggregate pores and microporous defects. By altering carbazole‐based backbones, pore size and pore connectivity are adjusted. The rigid and interconnected confinements restrict molecular rotation and vibration, enforcing consistent shapes and orientations. This enables the separation of solute molecules (≈1000 g mol^−1^) with linear and bulky shapes, achieving separation factors of up to 134. When pore size is reduced to angstrom scale (≈5 Å), molecular shape significantly influences organic liquid transport. The CMP membranes demonstrate all‐liquid phase separation of linear/branched alkane isomers (<100 g mol^−1^), enriching hexane to 63.35 mole% from equimolar isomer mixture and achieving permeance orders of magnitude higher than those of state‐of‐the‐art membranes.

## Graphical abstract

Conjugated microporous polymer membranes with narrowly distributed network pores are prepared by diffusion‐modulated electropolymerization. This approach precisely controls the monomer diffusion and coupling processes, regulating the crosslinking degree to prevent broad aggregate pores and microporous defects. The rigid and interconnected confinements restrict molecular rotation and vibration, enforcing consistent shapes and orientations. This enables the shape selective separation of molecules.

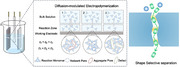

## 1 Introduction

Precisely separating individual molecular species from mixtures in a low‐energy and high‐efficiency mode is the long‐term goal pursued by scientists and engineers. This is particularly relevant for industrial applications that require the separation of complex mixtures containing molecules with similar sizes but different shapes (linear, block, dendritic, branch, etc.). One such example is crude oil, which comprises flexible linear and branched aliphatic hydrocarbons, and cyclic aromatic hydrocarbons.^[^ ^1^ ^]^ In addition, the precise separation of isomers, including alkane isomers, methylated aromatics (xylenes, benzene, and toluene),^[^ ^2^ ^]^ and divinylbenzene isomers (*m*‐divinylbenzene and *p*‐divinylbenzene),^[^ ^3^ ^]^ is of significant importance. Vapor separation, pervaporation, and adsorption have been widely investigated as potential methods for these separations in academic communities; however, most existing technologies depend on energy‐intensive thermal processes and complex procedures, leading to high operating costs.

Pressure‐driven membrane technologies offer energy‐efficient alternative for separation. The earliest pressure‐driven membranes were designed to differentiate molecules based on three‐dimensional size, considering parameters such as length, width, and height. However, relying solely on size proves problematic when dealing with molecules that have minimal size differences or unpredictable effective sizes, influenced by their flexibility, rotational dynamics, and random orientations.^[^ ^4^ ^]^ For instance, polyethylene glycol (PEG), a linear and flexible polymer, can alternate between stretched and coiled configurations depending on the environment. In addition, in the separation of organic isomers, where size differences are typically minimal (<0.1–0.3 nm), the random orientations of molecules in the mixture,^[^ ^5^ ^]^ further complicate size‐based separations. State‐of‐the‐art membranes face the challenges to efficiently differentiate such mixtures.^[^ ^6^ ^]^ Shape selectivity, also known as entropic selectivity, is a molecular discrimination mechanism found in microporous materials with rigid and confined pore structures.^[^ ^7^ ^]^ Within these rigid, interconnected transport channels, the freedom of movement for molecules, specifically their rotational and vibrational degrees of freedom, is significantly restricted. This reduction in mobility varies among molecules of different shapes as illustrated in Figure S1 (Supporting Information), which is translated into different entropic changes. As a result, it enables membranes to effectively recognize and separate molecules based on their shapes, addressing the limitations of traditional size‐based separation methods.

Achieving shape‐based separation requires precise control over the rigid and interconnected confinements within membranes to match the size and geometry of the molecules being separated. While traditional polymeric membranes are widely used, they are often less effective in distinguishing between molecules in organic solvents due to the poorly defined pore sizes and increased segmental motions of polymer chains, diminishing the potential entropic or shape selectivity.^[^ ^5^ ^]^ Conjugated microporous polymers (CMPs) are promising candidate materials due to their rigid π‐conjugated skeletons and permanent micropores. The pore size and structure of CMPs can be tailored at the molecular level.^[^ ^8^ ^]^ Formed via the irreversible reactions of their building blocks,^[^ ^9^ ^]^ CMPs feature exceptional chemical and thermal stability.^[^ ^9^ ^]^ They are less prone to structural relaxation in organic solvents,^[^ ^10^ ^]^ making them appealing for practical applications in solvent‐based systems. Recently, significant progress has been made in processing CMPs into functional membranes, demonstrating their potential in separating molecules and ions.^[^ ^10^, ^11^ ^]^ However, the potential of CMP membranes for shape‐selective separations remains largely unexplored.

The CMP membranes can be fabricated via electrochemical approach. This method does not require complex equipment and involves fewer harmful solvents and chemicals compared to traditional CMP membrane fabrication techniques, which typically rely on toxic reagents and precious metal catalysts.^[^ ^11^, ^12^ ^]^ The eletropolymerization process involves (1) diffusion of precursor molecules to the electrode surface, (2) oxidation of monomers into radical cations, and (3) coupling of radical cations into polymers. Both the diffusion and coupling processes influence the membrane structure and ultimately the membrane performance. The lack of precise control over these two processes often results in broadly distributed aggregate pores or microporous defects within the membranes. Achieving CMP membranes with narrowly distributed network pores through electropolymerization remains challenging, yet it is essential for producing membranes with high shape selectivity.

Herein, we report the design of CMP membranes with tailored rigid and interconnected confinements through diffusion‐modulated electropolymerization. By adjusting the viscosity (*η*) of the electrolyte solution, we controlled the monomer diffusion coefficient (*D*_0_) and the monomer replenishment during coupling process, thereby achieving an optimal crosslinking degree. This modulation effectively prevents the formation of aggregate pores and microporous defects, while promoting the formation of narrowly distributed network pores. In addition, varying the rigid backbones of carbazole‐based repeat units allows for the tuning of pore size and pore connectivity. The resulting CMP membranes, with uniform, rigid and interconnected pore channels, show great promise for shape‐based separations, particularly for solute molecules (≈1000 g mol^−1^) with both linear and bulky shapes. To further validate this concept, we investigated smaller organic solvent molecules and found that subnanometer changes in pore size leads to significant variations in organic solvent transport behaviors. When the pore size is reduced to the angstrom scale (≈5 Å), the CMP membranes enable effective all‐liquid phase separation of linear and branched alkane isomers (<100 g mol^−1^), enriching hexane to 63.35 mole% from an equimolar mixture, achieving permeance several orders of magnitude higher than current state‐of‐the‐art membranes. Our findings reveal that CMP membranes present significant potential in shape separation of these molecules, when the pore channels are properly engineered. This work is the first example of CMP membranes for shape‐selective molecular separations and sheds new light on value‐added separations through CMP membranes.

## 2 Results and Discussions

### 2.1 Diffusion‐Modulated Electropolymerization for Crosslinking Degree Control in CMP Membranes

**Figure 1:**
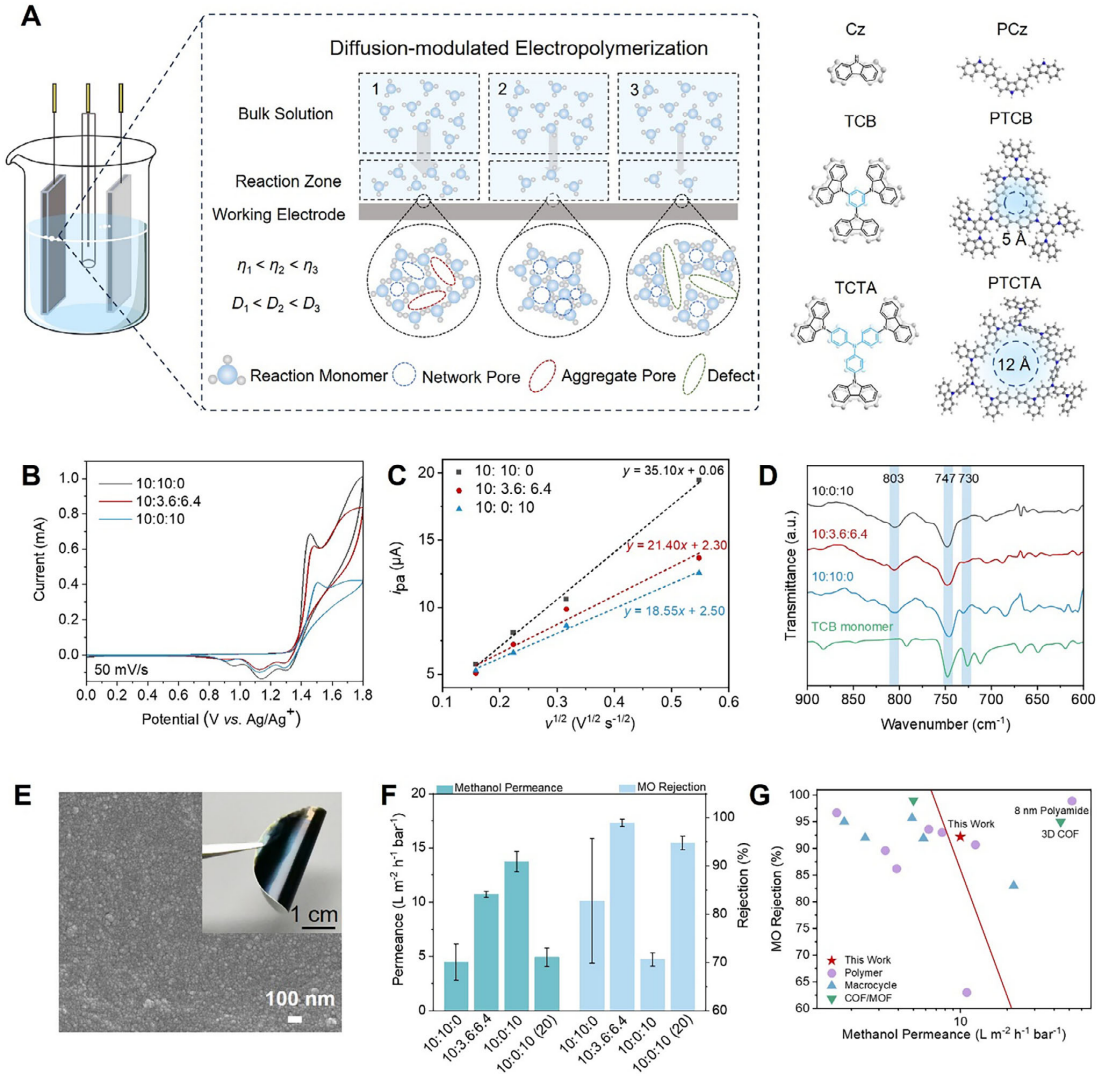
Fabrication and characterization of CMP membranes. A) Schematic illustration of diffusion‐modulated electropolymerization using three carbazole‐based monomers (Cz: carbazole, TCB: 1,3,5‐tris(N‐carbazolyl)benzene, and TCTA: tris(4‐carbazoyl‐9‐ylphenyl)amine) for fabricating CMP membranes with varying microstructures. B) The first CV curve of TCB electropolymerized in three different electrolyte solutions on indium tin oxide (ITO) glass. C) Anodic peak current (*i*_pa_) as a function of the square root of the scan rate (*v* ^1/2^). The *i*_pa_ values are derived from the cyclic voltammetry (CV) curves in Figure S7 (Supporting Information) at 1.08 V. D) Fourier‐transform infrared (FTIR) spectroscopy spectra showing the TCB monomer and PTCB films electropolymerized in three different electrolyte solutions. E) Scanning electron microscope (SEM) image of a PTCB membrane electropolymerized in electrolyte solution with DCM: ACN: PC ratio of 10: 3.6: 6.4 (*v*/*v*/*v*), DCM: dichloromethane, ACN: acetonitrile, PC: propylene carbonate. The inset displays a digital photograph of the PTCB membrane. F) Pure methanol permeance and methyl orange (MO) rejection performance of PTCB membranes fabricated by eight scan cycles. Data labeled as “(20)” indicate performance with the scan cycles increased to 20. The feed solution for rejection test contains 50 ppm MO in methanol. Error bars represent standard deviations for three different measurements for each data point. G) Comparison of the separation performance of tailored PTCB membranes with those of state‐of‐the‐art membranes reported in the literature.

The pore size and pore connectivity of the membranes were tailored by choosing three carbazole‐based monomers with single or multiple carbazole units (**Figure** **1**A). Carbazole (Cz) possessing two electrochemical active sites forms membranes with densely packed linear polymer chains, which is denoted as PCz. On the contrary, 1,3,5‐tris(N‐carbazolyl)benzene (TCB) and tris(4‐carbazoyl‐9‐ylphenyl)amine (TCTA) with multiple carbazole units arranged around a core unit present more than two electrochemical active sites. The multi‐arm alignment makes it possible to form 3D porous skeletons with better pore connectivity compared to PCz (Figure S2, Supporting Information). TCB has a smaller core unit compared to TCTA, resulting in smaller pores in the PTCB membrane than those in the PTCTA membrane. The electrosynthesis of PCz, PTCB, and PTCTA films was conducted by multicycled cyclic voltammetry (CV) (Figure S3, Supporting Information) in a three‐electrode electrochemical cell, where carbon nanotube (CNT) supporting membrane, titanium metal plate and Ag/AgCl electrode were used as the working, counter, and reference electrodes, respectively. The electropolymerization mechanism of Cz, TCB and TCTA is presented in Figure S4 (Supporting Information).

Achieving highly crosslinked polycarbazole structures through electropolymerization remains challenging. Once coupling occurs at the 3‐position of the carbazole groups, subsequent coupling at the 6‐position becomes progressively more difficult,^[^ ^13^ ^]^ resulting in a low degree of crosslinking (Figure S5, Supporting Information). Therefore, broadly distributed aggregated pores rather than narrowly distributed network pores are created within the membrane. Controlling the degree of crosslinking requires precise regulation of the coupling process, which is largely influenced by the diffusion coefficient (*D*_0_) of the monomer molecules. Variations in *D*_0_ affect the monomer concentration near the electrode surface (Figure 1A). At a higher *D*_0_, faster monomer replenishment promotes preferential 3‐position coupling. In contrast, we envisage the coupling process is more controlled at a lower *D*_0_, leading to membranes with higher crosslinking degree due to slower monomer replenishment and more complete coupling reactions. The viscosity of the electrolyte solution significantly affects diffusion, with higher viscosities hindering this process. To modulate the value of *D*_0_, the electrolyte viscosity was adjusted by replacing dichloromethane (DCM) and acetonitrile (ACN) with propylene carbonate (PC), a more viscous solvent.^[^ ^14^ ^]^ In studies on the PTCB membranes, three different electrolyte solutions were used: (1) DCM: ACN: PC = 10: 10: 0 (*v*/*v*/*v*), (2) DCM: ACN: PC = 10: 3.6: 6.4 (*v*/*v*/*v*), and (3) DCM: ACN: PC = 10: 0: 10 (*v*/*v*/*v*), with viscosities at 25 °C of 0.48, 0.80, and 1.12 mPa s, respectively. The CV curves showed carbazole oxidation peaks at 1.08 and 1.45 V (Figure 1B). The increased viscosity led to reduced oxidation currents, indicating a lower quantity of monomers reacting at the electrode surface and consequently thinner electropolymerized films (Figure S6, Supporting Information). According to the Randles–Sevcik equation (298 K)^[^ ^15^ ^]^

(1) $$i_{p}=2.69\times 10^{5}An^{3/2}D_{0}^{1/2}C_{0}v^{1/2}$$

where *i*_p_ is the peak current, *A* is the electrode surface area, *n* is the number of electrons transferred, *D*_0_ is the diffusion coefficient, *C*_0_ is the monomer concentration, and *v* is the scan rate. The linear relationship observed between the anodic oxidation peak current and the square root of the scan rate (Figure 1C) indicates that the electrode reaction is diffusion‐controlled. A steeper slope corresponds to a higher *D*_0_, which diminishes with increasing viscosity. Fourier‐transform infrared (FTIR) spectroscopy analysis confirms these chemical structural distinctions (Figure 1D). The characteristic peaks of TCB monomers at 730 and 747 cm^−1^, corresponding to C─H bending vibrations in the 1,2‐disubstituted benzene rings of the carbazole groups, weakened with decreasing *D*_0_. This reduction indicates fewer residual carbazole groups, as most carbazole groups coupled at positions 3 and 6 to form crosslinked structure. The appearance of a new peak at 803 cm^−1^ confirmed successful carbazole crosslinking. Lower *D*_0_ promotes more complete monomer reactions on the electrode, resulting in films with higher crosslinking degree, as well as smoother and denser surfaces (Figure 1E and Figure S8, Supporting Information). Low‐field nuclear magnetic resonance (LF NMR) measurements were conducted to analyze the transverse relaxation time (T_2_) of hydrogen protons in the methanol molecules within the membrane. This analysis provides valuable insights into the pore size distribution and the mobility of solvent molecules across different regions of the membrane.^[^ ^16^ ^]^ As shown in Figure S9 (Supporting Information), the fastest relaxation fraction, T_2b_ at 0.01 ms, corresponds to methanol molecules that are strongly bound to the membrane, representing the “strongly bound methanol.” The network pore structure is reflected by the T_21_ peak, with a relaxation time of less than 10 ms, corresponding to subnanometer pores in the range of ≈5 Å.^[^ ^17^ ^]^ The T_22_ peak, with a relaxation time around 10 ms, is attributed to pores of ≈1 nm.^[^ ^17^ ^]^ It was observed that the highest *D*_0_ (10:10:0) condition results in a lower proportion of network pores and a broader pore size distribution. This is due to the lower degree of crosslinking, which leads to the formation of aggregated pores. When *D*_0_ is reduced (10:3.6:6.4), more network pores are generated. However, when *D*_0_ is too low (10:0:10), increased crosslinking leads to more microporous defects, as indicated by the stronger T_23_ peak. This peak appears with a broad distribution in the 100–300 ms time domain and reflects the presence of microporous defects, which may contribute to the interconnectivity of the membrane. A certain proportion of microporous defects can create pathways between the defined pores, thereby enhancing the overall connectivity of the network. This interconnectivity is crucial for molecular transport, as it facilitates the movement of molecules through the membrane. However, an excessive amount of microporous defects can negatively impact the membrane's ability to reject small molecules with subnanometer sizes. Notably, there is no peak at 1000 ms or above, which is attributed to the free space in the membrane, indicating that large structural defects are absent in the three PTCB membranes.

Performance tests for solutes with different molecule weights in methanol are shown in Figure 1F and Figure S10 (Supporting Information). While these CMP membranes demonstrate similar rejection for larger solutes, membranes prepared with different *D*_0_ present different permeance and rejection to small solute methyl orange (MO, MW = 327 g mol^−1^). When *D*_0_ is too high (i.e., under the condition of 10:10:0), the resulting broad distribution of aggregated pores leads to an 82.7% rejection of MO. In contrast, when *D*_0_ is optimized (10: 3.6: 6.4), the MO rejection increases to 98.9% with enhanced permeance. This improvement is attributed to the formation of more network pores and thinner films. However, when *D*_0_ is too low (10: 0: 10), the high crosslinking degree leads to overly rigid polymer chains and the formation of dispersed crosslinked structures, introducing microporous defects that compromised MO rejection. Increasing thickness by adding more CV cycles could not completely offset these microporous defects and significantly reduce permeance. Overall, the viscosity of the electrolyte can modulate *D*_0_, which in turn affects the rate of monomer replenishment during coupling process, thereby enabling precise control over the degree of crosslinking. The PTCB membrane prepared at optimal electrolyte viscosity exhibits a defect‐free, moderately crosslinked structure that effectively balance small molecule rejection and permeability, thereby overcoming the trade‐off in membrane performance (Figure 1G).

This approach also enables the synthesis of defect‐free PCz and PTCTA membranes. Details of optimal experimental conditions and membrane characterizations are provided in Text SI (Supporting Information). Nitrogen physisorption at low pressure (Figure S14, Supporting Information) confirms the microporosity of CMP films forms with 3D porous skeletons, that provides accessible channels for molecular transport. In addition, PTCB membranes presents smaller pore size (≈5 Å) compared to that of PTCTA membranes (≈12 Å) (Figure S15, Supporting Information), which may be due to the smaller rigid core unit of TCB than that in TCTA monomer. In contrast, the PCz membranes with densely packed linear polymer chains exhibits an ill‐interconnected pore structure, due to its nearly planar structure, which promotes parallel intermolecular π–π stacking.^[^ ^18^ ^]^ Our LF NMR measurements (Figure S16, Supporting Information) show that Cz membranes lack the peak at 100 ms, indicating a less interconnected pore network compared to those formed with other monomers such as TCTA and TCB with non‐coplanar, rigid, and twisted structures. The as‐synthesized CMP membranes formed on the CNTs support are flexible and robust (inset of Figure 1D). Moreover, as shown in Figure S17 (Supporting Information), the Young's modulus of the PTCB membrane as high as 4.3 GPa. This high rigidity of the membrane is beneficial for shape‐selective separation involving organic solvents.

### 2.2 Shape‐Selective Separation of Solutes Mixtures

**Figure 2:**
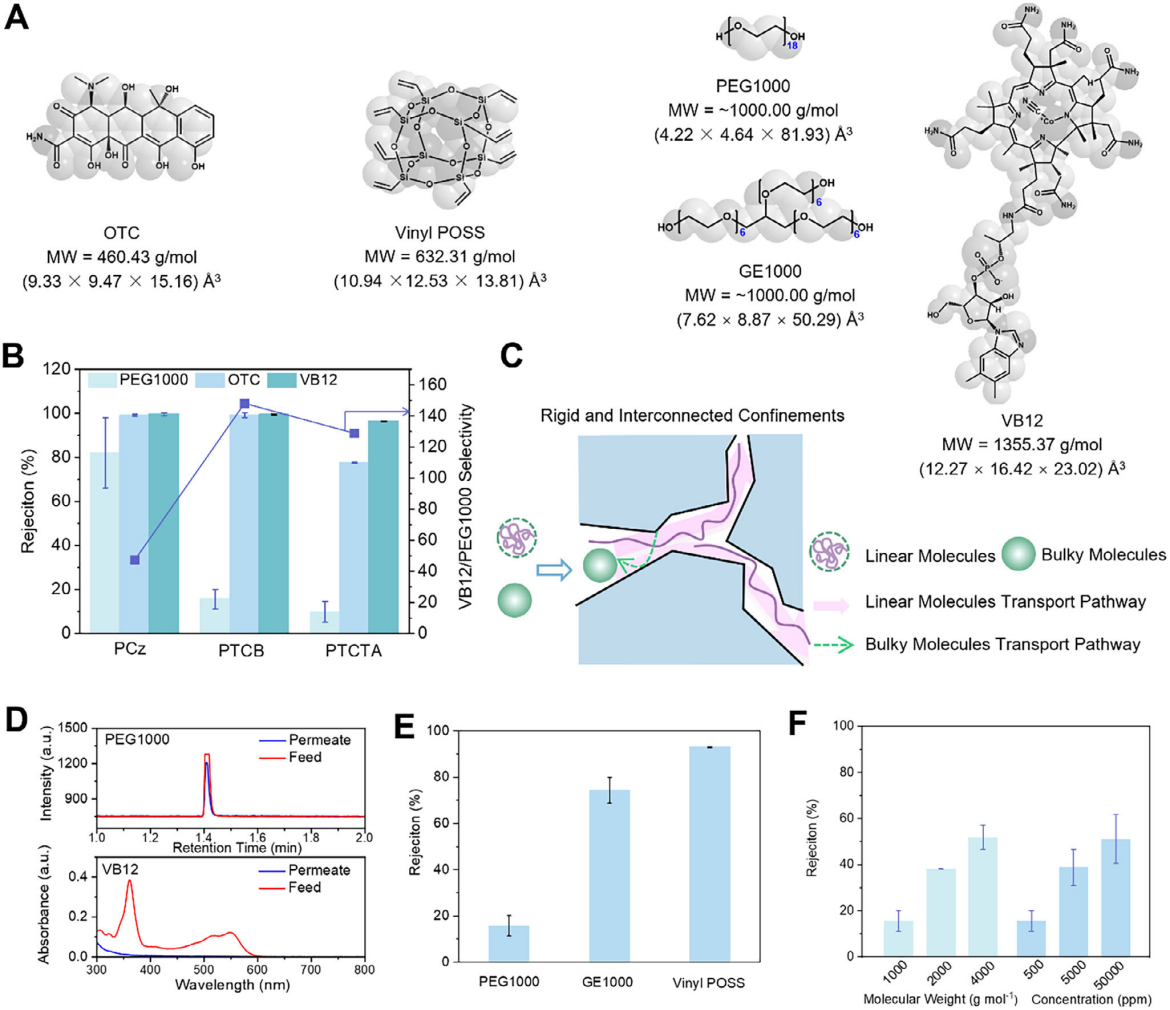
Shape‐selective transport of solutes in ethanol through CMP membranes. A) Molecular structure of oxytetracycline (OTC), vinyl polyhedral oligomeric silsesquioxane (Vinyl POSS), polyethylene glycol 1000 (PEG 1000), glycerol ethoxylate 1000 (GE1000), and (Vitamin B_12_) VB12. It is noted that only the repeating units of PEG1000 and GE1000 are shown. The 3D size of the solutes was estimated based on methods from our prior research,^[^ ^12^, ^23^ ^]^ the dimensions are listed in the order from the smallest length side to the largest length side of the box that contains the molecules. B) Rejection of PCz, PTCB, and PTCTA membranes toward various solutes in ethanol. The feed concentration was 500 ppm. C) Schematic representations of long‐chain molecules and bulky molecules passing through the confined channels of CMP membranes. D) High‐performance liquid chromatography intensity (HPLC) curve and ultraviolet–visible (UV–vis) absorbance curve of the feed and permeate after filtration through PTCB membrane. The feed solution was 50 ppm PEG1000 and 50 ppm VB12 in ethanol. The rejections to PEG100 and VB12 are 38.47% and 99.54%, respectively. E) Rejection of PTCB membrane to solutes with linear, branched, and globular structure in ethanol. The feed concentration was 500 ppm. F) Rejection of PTCB membrane toward PEG molecules with various molecular weight in ethanol at the concentration of 500 ppm, and toward PEG1000 with various concentration in ethanol. Error bars represent standard deviations for three different measurements for each data point.

The shape sieving capability of three CMP membranes was first investigated by dead‐end cell, a widely adopted technique for evaluating membranes performance.^[^ ^1^, ^19^ ^]^ Solutes with distinct molecular structures have been used, including flexible linear‐chain polyethylene glycol 1000 (PEG1000, MW ≈ 1000 g mol^−1^), bulky oxytetracycline (OTC, MW = 460.46 g mol^−1^), and Vitamin B_12_ (VB12, MW = 1355.37 g mol^−1^) as shown in **Figure** **2**A and Table S1 (Supporting Information). The transport behavior of molecules is strongly affected by confined channels. PTCB and PTCTA membranes exhibit interesting features in discriminating solute molecules based on their shapes. Flexible linear‐chain PEG1000, with equivalent Stokes diameter of 1.57 nm correlating with its molecular weight,^[^ ^20^ ^]^ is able to permeate through the PTCB and PTCTA membranes at the rejections below 20%, while the bulky OTC and VB12 molecules with smaller or comparable molecular weights are effectively rejected (mostly >90%) (Figure 2B and Table S2, Supporting Information). This contrasts with that of PCz membrane presenting much higher rejection for PEG1000, OTC, and VB12 solutes. The ability of PTCB and PTCTA membranes to separate PEG1000 from bulky molecules may arise from their rigid and interconnected transport channel. PEG, due to their inherent flexibility, exhibits unique behaviors under flow, which are distinct from rigid molecules. As illustrated in Figure 2C, the linear PEG molecules tend to adopt self‐entangled globular conformations, with a random orientation in the bulk. Under pressure‐driven flow, shear forces play a significant role in aligning polymer chains along the flow direction,^[^ ^21^ ^]^ these shear forces, acting at PEG‐pore wall interface, induce deformation, stretching, and reorientation of the chains. As a result, the PEG adopts a more extended conformation within the narrow confines of the pores to minimize steric hindrance.^[^ ^4^ ^]^ This conformational adjustment enables PEG chains to pass more easily through the membrane, enhancing selective permeation.^[^ ^22^ ^]^ On the other hand, the bulky molecules with less flexibility are substantially retained due to the restricted access into the confined micropores. Consequently, the ideal VB12/PEG1000 selectivities for PTCB and PTCTA are 148.1 and 128.8, respectively. In contrast, the PCz membrane with ill‐interconnected pore structure, does not exhibit significant shape selectivity. Furthermore, we demonstrated the effective separations of linear molecules from mixtures containing bulky molecules. PEG1000 and VB12 were mixed in ethanol as the feed. PTCB membranes demonstrate a high VB12/PEG1000 selectivity of 134 that is comparable to the ideal selectivity, confirming its pronounced shape selectivity (Figure 2D). Mixture separation was also performed using cross‐flow filtration (Figure S18, Supporting Information), and our results showed similar selectivity compared to dead‐end mode. In addition, long‐term testing is essential to rule out adsorption effects and demonstrate the stability of the membranes under cross‐flow mode over time (Figure S19, Supporting Information).

To further evaluate the shape‐selective separation ability of PTCB membranes, we measured the rejection to solutes with other geometries including branched and globular structures. As shown in Figure 2E, the membranes again present effective differentiation toward shapes. The rejection to linear PEG1000 is 15.6%, whereas branched glycerol ethoxylate 1000 (GE1000, MW = 1000 g mol^−1^) is retained by 74.3%. In the meantime, the rejection to the rigid and globular vinyl polyhedral oligomeric silsesquioxane (Vinyl POSS, MW = 632.31 g mol^−1^) reaches 93.0%, much higher than that for linear PEG1000. The membrane presents lower rejection to branched GE1000 than POSS, though bearing a higher molecular weight. This is consistent with earlier discussion that soft polymer chains self‐adapt in the pore channel to pass through it. The results suggest that the transport of solute molecules through the micropore is impacted by their shapes and flexibilities. We also explored the transport behavior of PEG molecules with different molecular weights and concentrations. As shown in Figure 2F, PTCB membrane exhibits an increased rejection to PEG molecules as the molecular weight increases, which might be associated with the greater entropy barriers for stretching due to their longer chain lengths and increased distortion. It is also observed that increasing concentration of PEG molecules in the solvent lead to higher rejection. As is known, the polymer chains may start to overlap and entangle with each other at higher polymer concentrations. While PEG molecules are linear and pass through the confined pores in extended, aligned conformation, the increase in chain length and concentrations results in increased distortion and higher entropic barriers for the conformational transition from random‐coil to extended shape. Even that in all scenarios, the rejection remains lower than 60%, confirming the selective transport of linear molecules across the microporous PTCB membrane.

**Figure 3:**
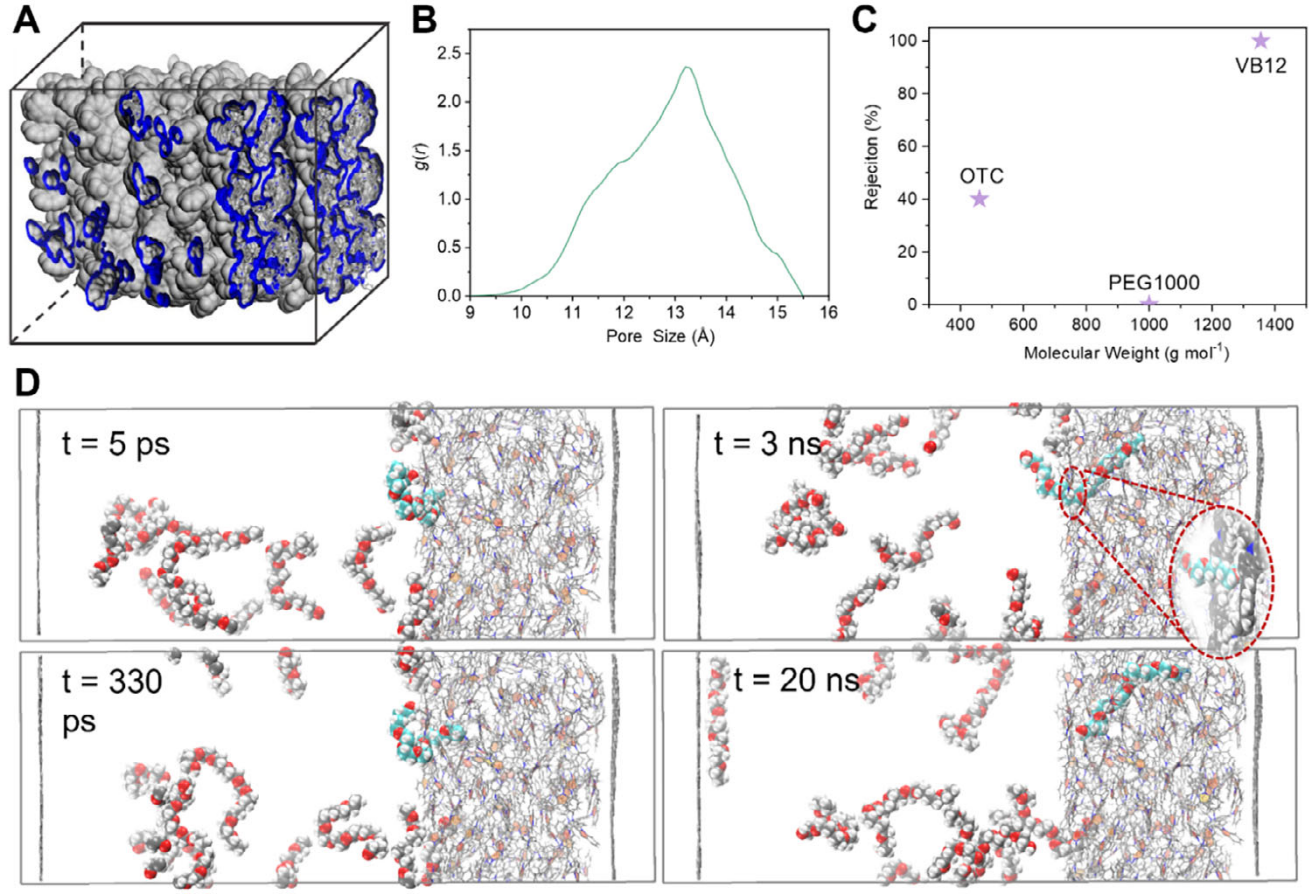
Simulation of PTCTA membrane and molecular transport. A) Simulated 3D view of the PTCTA membrane; gray shading and blue area represent free volume and Connolly surface in the membrane, respectively. B) Pore size distributions of PTCTA membrane. C) Rejection of PTCTA membrane toward various solutes in ethanol, OTC: oxytetracycline, VB12: Vitamin B_12_ and PEG1000: polyethylene glycol 1000. D) Snapshots for the diffusion of PEG chains across PTCTA matrix.

The microporous structure of CMP membranes was constructed by molecular dynamics (MD) simulations, represented by PTCTA membrane. Figure S20 (Supporting Information) presents TCTA monomer, dimer, and trimer. Due to the advantageous cooperativity effect in the trimer of TCTA (−2.45 kJ mol^−1^ at M06‐2X/6‐311+G(d) level), TCTA trimer contributes to the pore formation and was considered as the structural unit of the PTCTA membrane. Interconnected pore structure is confirmed by the simulation (**Figure** **3**A). The calculated pore size distribution of PTCTA membrane was ≈13 Å (Figure 3B), which is comparable to the experimental result (≈12 Å). To evaluate the shape selectivity of PTCTA membrane, the rejection of PTCTA membrane to PEG chain, OTC and VB12 in ethanol was simulated in the system as shown in Figure S21 (Supporting Information). All PEG molecules penetrate through the PTCTA membranes (Figure 3C), while VB12 is completely rejected. The rejection to OTC falls in the middle, which is consistent with experimental results. In order to gain insights into the transport of PEG chains through the membrane, we performed MD simulation to investigate the insertion and diffusion behaviors of the PEG chains (Figure 3D). Initially, the PEG chains are randomly dispersed in the ethanol phase, self‐tangled or inter‐tangled. After 5 ps, three PEG chains near the interface region approach the PTCTA membrane surface. At 300 ps, one end of a PEG chain enters the membrane pore, while the other end remains bent and dangling outside the pore. The inset figure illustrates PEG chains transport across the pores of an outset PTCTA trimer. At 3 ns, more PEG chains start to move inward into the pores and by 20 ns, the entire PEG chain crawls further into the PTCTA membrane matrix, demonstrating the conformational adapting behavior of PEG chains within the interconnected pore channel of CMP membranes.

### 2.3 Transport Mechanisms of Organic Solvents in CMP Membranes

**Figure 4:**
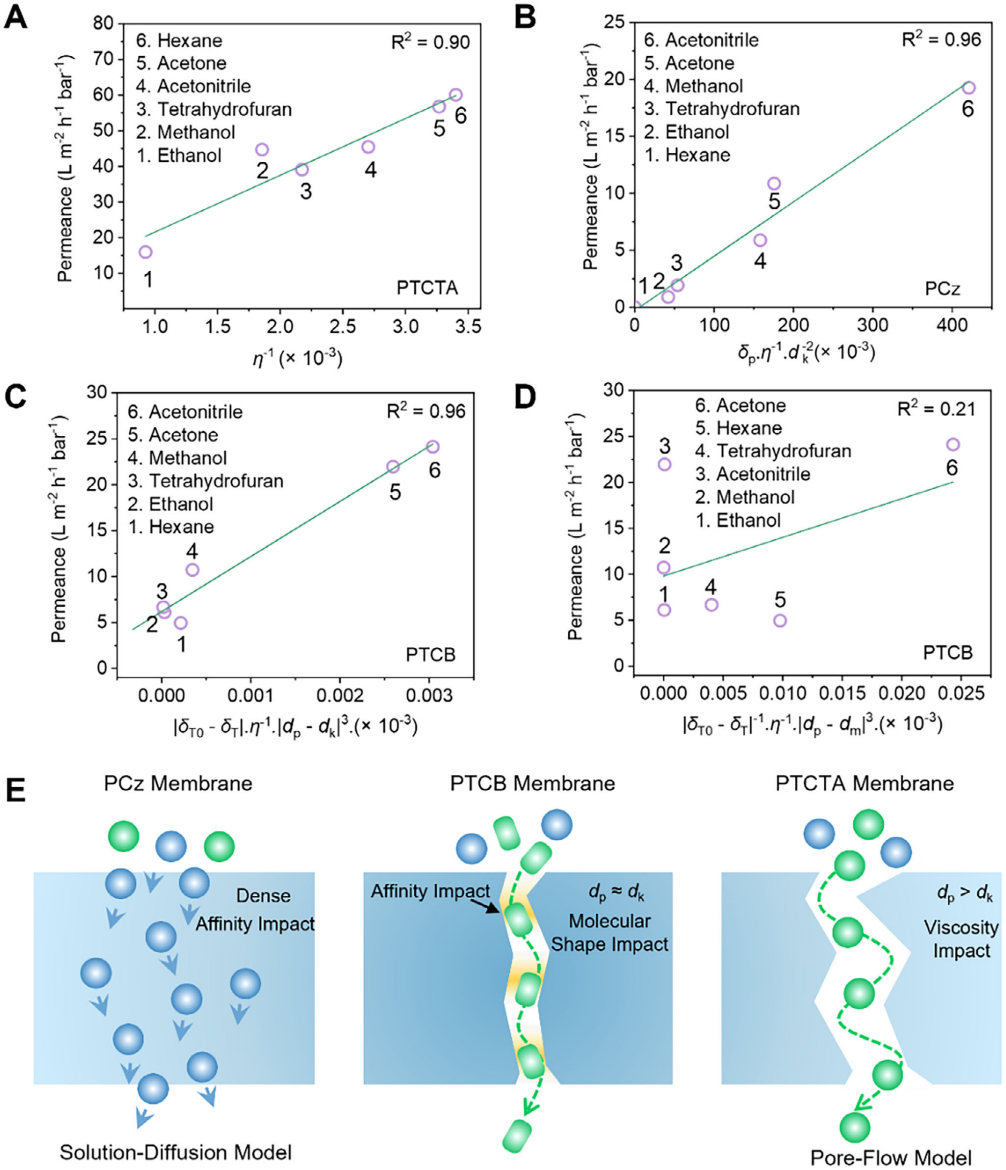
Organic solvent transport through CMP membranes. A) Solvent permeance of the PTCTA membranes against inverse of solvent viscosity. B) Solvent permeance of the PCz membranes as a function of model parameter combining Hanson solubility parameter, solvent viscosity, and solvents. C) Solvent permeance of the PTCB membrane as a function of model parameters combining solvent viscosity, pore size, and total Hanson solubility parameter of PTCB membrane, and kinetic diameter and total Hanson solubility parameter of solvents. D) Solvent permeance of the PTCB membranes as a function of model parameter combining solvent viscosity, pore size, and total Hanson solubility parameter of PTCB membrane, and molar diameter and total Hanson solubility parameter of solvents. E) A schematic illustrating the solvent transport mechanism of three CMP membranes.

Given that the pore sizes and connectivity are the main difference among the three carbazole‐based CMP membranes, we performed systematic study on the permeation of a series of organic solvents through the CMP membranes to unravel their transport mechanisms. Transport models are typically defined by model parameters that depend on structural and physicochemical properties of membrane and solvent.^[^ ^24^ ^]^ These properties include membrane pore size/free volume, solvent viscosity (*η*), Hanson solubility parameter (HSP), and the size of solvent molecules which is represented by either kinetic diameter (*d*_k_) or molar diameter (*d*_m_), among others. PTCTA membrane with pore size of ≈1.2 nm, adheres to the pore‐flow model, as a linear relationship (*R* ^2^ = 0.90) between permeance and inverse of solvent viscosity 1/*η* is seen (**Figure** **4**A). Detailed discussions about the pore‐flow model for PTCTA membranes can be found in Text SII (Supporting Information). For PCz membranes with linearly packed polymer chains and ill‐connected channels, plotting the solvent permeances across PCz membranes against *η* ^−1^ and *δ_p_ η* ^−1^ *d*_k_^−2^ gives *R* ^2^ of 0.09 and 0.96, respectively (Figure S23A, Supporting Information, and Figure 4B). This suggests that the transport of solvent through PCz membranes largely deviates from viscous flow behavior and is influenced by HSP (*δ_p_*) and kinetic diameter (*d*_k_), aligning with solution‐diffusion model. It is also noticed that despite of thin selective layer of ≈72 nm (Figure S24A, Supporting Information), the hexane permeance was below the detection limit for PCz, making it difficult to test the permeation of nonpolar molecules with larger size.

The solvent transport across PTCB membranes with small pore size of ≈5 Å do not follow either the typical pore flow model or solution‐diffusion model, according to the plots of solvent permeances against *η* ^−1^ and *δ_p_ η* ^−1^ *d*_k_^−2^ give *R* ^2^ of 0.19 and 0.64, respectively (Figure S25, Supporting Information). To understand where the deviation comes from, we first plotted the product of permeance and viscosity against solubility parameter of organic solvents. As shown in Figure S26 (Supporting Information), the product of permeance and viscosity is not constant when plotted against the total HSP (*δ*_T_) value of the solvent. Molecular transport is a function of solubility in an interesting way, such that the total HSP value corresponding to peak of permeance × viscosity is close to the total HSP of PTCB membrane (*δ*_T0_), which is 20.3 MPa^1/2^ (Table S3, Supporting Information). That indicates the total HSP value difference between the membrane and solvent affect the transport in the PTCB membrane. The strong affinity between solvents and PTCB membrane, which is reflected in the closeness of their solubility parameters, results in high transport rates of the solvents. This characteristic can be leveraged in solvent separation, such as the application in acetone‐butanol‐ethanol fermentation^[^ ^25^ ^]^ (Text SIII, Supporting Information). Based on this observation, it can be inferred that the size difference between pore and the solvent molecules impacts the transport in this small confinement, thus, a correlation below is established:

(2) $$P\propto {\left|\delta_{T_{0}}-\delta_{T}\right|}^{-1}\times \eta^{-1}\times {\left|d_{p}-d_{k}\right|}^{3}$$

where *P* is the solvent permeance and *d*_p_ is the pore size of PTCB membrane. As presented in Figure 4C, this equation well demonstrates the phenomenological transport in PTCB membrane. It highlights the significant role of total HSP value difference, and the difference between kinetic diameter of solvent molecules (*d*_k_) and the pore size of PTCB membrane in governing solvent permeance. It is noted that, from a physics perspective, cubed dependence of size difference (|*d*_p_−*d*_k_|^3^) is typically the sphere volume difference between a molecule and the pore that governs transport phenomena. This cubed dependence indicates a more pronounced effect of diameter differences on transport rates. Notably, when molar diameter (*d*_m_) was applied in the model fitting, considerably lower *R* ^2^ of 0.21 were observed (Figure 4D). This discrepancy arises because kinetic diameter considers both the size and shape of the molecules and reflects the smallest effective dimension of the molecule,^[^ ^26^ ^]^ whereas the molar diameter assumes that each molecule can be represented as an equivalent sphere with a diameter of *d*_m_, which is a measure for the size of the molecule and may not adequately account for their actual molecular shapes.^[^ ^27^ ^]^ This finding highlights the influence of molecular shape of organic solvent on their transport within the PTCB membranes. Overall, the discussions have revealed that subnanometer changes to the pore channel of CMP membranes result in significant differences in transport behavior of organic liquids based on molecular affinity and shapes (Figure 4E).

### 2.4 Shape‐Selective Separation of Organic Liquid Mixtures

**Figure 5:**
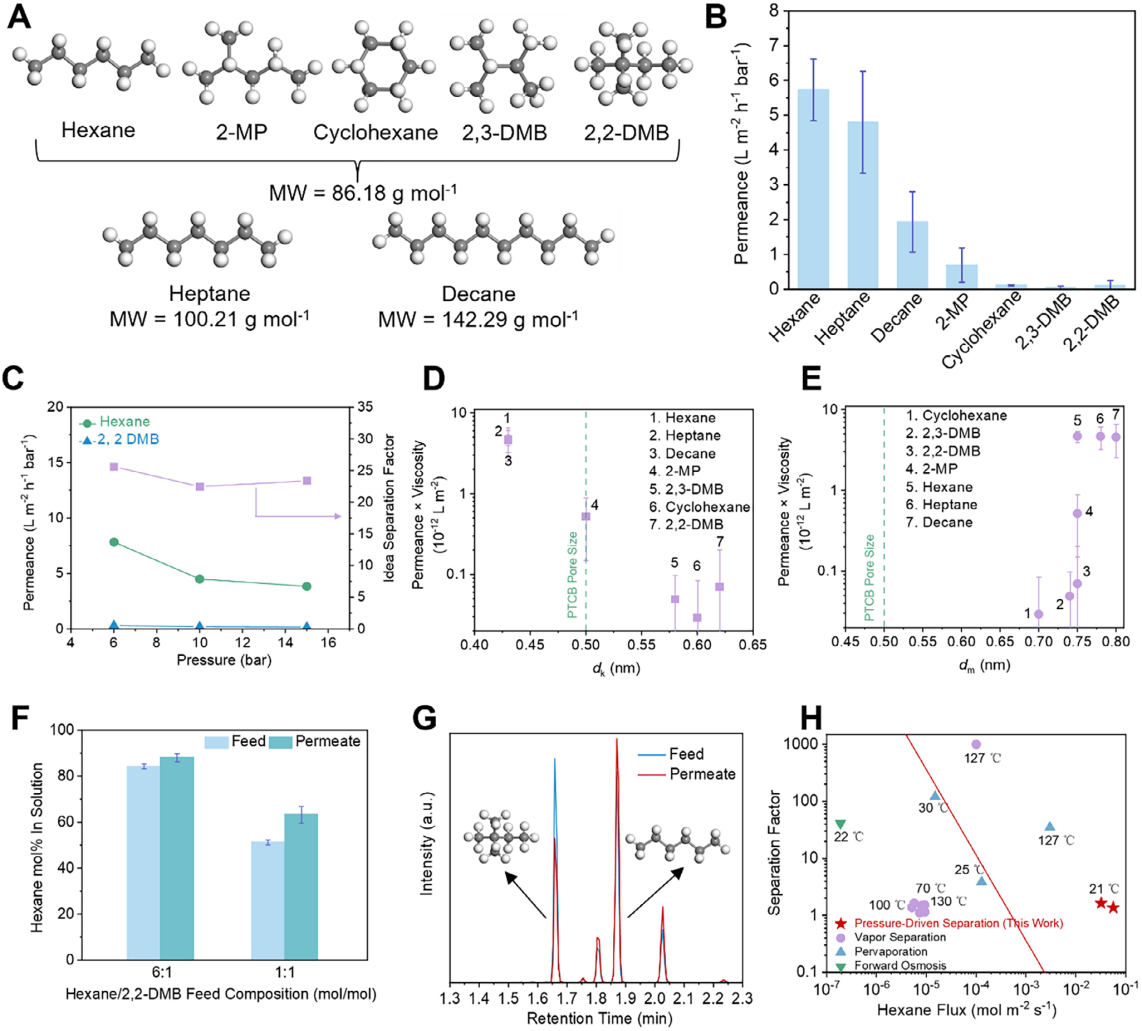
Separation of low‐molecular‐weight alkanes mixtures. A) Molecular structure of low‐molecular‐weight alkanes with different shapes: linear hexane (C6), heptane (C7), and decane (C10), mono‐branch 2‐methylpentane (2‐MP), dibranch 2,3‐dimethylbutane (2,3‐DMB), and 2,2‐dimethylbutane (2,2‐DMB), cyclic cyclohexane. B) Single‐component permeation of low‐molecular‐weight alkanes through PTCB membranes. C) Single‐component permeation of hexane and 2,2‐DMB and hexane/2,2‐DMB ideal selectivity of PTCB membranes under different transmembrane pressures. Product of permeance and viscosity of different solvents as the function of D) kinetic diameter and E) molar diameter of alkanes for PTCB membranes. Error bars represent standard deviations for three different measurements for each data point. F) Hexane compositions in permeate when using 6:1 (mol/mol) and 1:1 (mol/mol) hexane/2,2‐DMB mixtures. Noted that the impurities 3‐methyl pentane and methylcyclopentane presenting in hexane were omitted from the calculations. G) Gas chromatograms (GC) of the feed and permeate through PTCB membranes. The feed was 1:1 (mol/mol) hexane/2,2‐DMB mixture. The peaks observed in (G), from left to right, correspond to 2,2‐DMB, 3‐methyl pentane, n‐hexane, and methylcyclopentane. H) Hexane/di‐branched isomer separation factor of the state‐of‐the‐art membranes as a function of hexane flux.

The separation of hexane isomers is crucial in various chemical production processes, including octane upgrading and the manufacturing of chemicals and pharmaceuticals, where high‐purity isomers are required. Separation of hexane isomers is technically demanding process because of their close physicochemical properties, including molecular weight, viscosity, boiling point, polarity, and size different (<0.1–0.3 nm).^[^ ^7^ ^]^ This makes molecular shape as the primary handle available for their discrimination.^[^ ^28^ ^]^ Hexane has a linear structure, while its isomers are either branched or cyclic (**Figure** **5**A). To further validate the concept of shape‐selective separation, we investigated whether CMP membranes could achieve shape selectivity for these small molecules using the PTCB membranes with a favorable pore size of ≈0.5 nm, which falls within the range of the kinetic diameter of hexane isomers. Figure 5B presents the single‐component permeation measurements of seven C6–C10 alkanes across PTCB membranes. The permeance of linear hexane through PTCB membrane is 4.96 L m^−2^ h^−1^ bar^−1^, whereas its branched isomers 2,3‐dimethyl butane (2,3‐DMB), and 2,2‐dimethyl butane (2,2‐DMB) as well as cyclohexane exhibit considerably lower permeances (<0.1 L m^−2^ h^−1^ bar^−1^). The rigid and interconnected confinements in PTCB membranes enhance the distinction between different molecules based on their shapes, which can then be recognized by the pore size of the membranes. Linear alkanes quickly permeate through the membrane due to their smallest effective dimension, i.e., their cross‐section diameter compared to membrane pores, while the transport of branched and cyclic isomers with bulkier structures, is largely confined even at high transmembrane pressure, presenting ideal selectivity between hexane and 2,2‐DMB up to 25 (Figure 5C). Notably, alkanes with greater molecular weight, such as heptane (C7, 100.21 g mol^−1^) and decane (C10, 142.29 g mol^−1^), exhibit higher permeances than the branched hexane isomers (C6, 86.18 g mol^−1^), attributed to their linear structure (Figure 5B). It is noted that, though the molecular size of long‐chain alkanes can vary significantly due to their high flexibility and rotational dynamics, when the size of the confining space is reduced to match the confined molecules, the rotational and vibrational motion of the confined molecules will be noticeably impeded,^[^ ^29^ ^]^ thereby facilitating a hyperloop‐like diffusion of these long‐chain molecules.^[^ ^30^ ^]^ Given the similar viscosity and HSP (TableS3, Supporting Information) of the alkane solvents being tested, when plotted against solvent size, as expected, the product of permeance and viscosity displays pronounced decreasing trend with increasing *d*_k_ (Figure 5D). Yet, this correlation becomes less discernible when aligned against *d*_m_ (Figure 5E). All these findings suggest that PTCB membranes possess the potentials for shape‐selective separations of hexane isomers.

To investigate the selective transport of hexane isomers in mixture feed, 6:1 and 1:1 (mol/mol) hexane/2,2‐DMB feed mixture were filtered through PTCB membrane (Figure 5F), Mixtures of hexane and 2,2‐DMB are effectively separated without phase changes under ambient conditions, yielding an enrichment up to 63.35 mole % of hexane from an equimolar feed. Notably, 2,2‐DMB permeates 5.76 and 14.05 times faster in 6:1 (mol/mol) and 1:1 (mol/mol) hexane/2,2‐DMB mixtures, respectively, while hexane permeates at a lower rate of 0.75 and 0.43 times, compared to their rates in single components, resulting in lower selectivities than the ideal selectivity obtained from single‐component permeation results. This indicates strong coupling effects in binary mixture feeds.^[^ ^7^ ^]^ Despite this, the confinement effect is dominant compared to the interactions between the hexane and isomer, resulting in higher hexane concentrations in the permeate compared to the initial feed (Figure 5G). Moreover, with abundant and interconnected uniform pores, PTCB membranes exhibit order‐of‐magnitude enhancement in hexane flux compared to state‐of‐the‐art membranes utilized in pervaporation, vapor separation, or forward osmosis (Figure 5H and Table S4, Supporting Information). Notably, the overall energy intensity for pressure‐driven membranes is significantly lower than processes involving phase changes such as vapor separation and pervaporation.^[^ ^31^ ^]^

## 3 Conclusion

In summary, this study addressed the challenging task of separating molecules with similar sizes but different shapes using CMP membranes with confinements engineered by diffusion‐modulated electropolymerization and monomer design. By controlling the monomer diffusion coefficient (*D*_0_) and regulating monomer replenishment during the coupling process, we achieved an optimal degree of crosslinking, effectively mitigating the formation of aggregate pore and microporous defects. By tuning carbazole‐based backbones, we adjusted pore size and pore connectivity. The rigid and interconnected confinements effectively restricted molecular rotation and vibration, ensuring consistent shapes and orientations during shape‐selective separations. A high separation factor of 134 between mixed solute molecules (≈1000 g mol^−1^) with linear and bulky structures was achieved. Furthermore, when the pore size is reduced to angstrom‐scale confinement (≈5 Å), the shape of the organic solvent molecules significantly influences their transport. We achieved effective liquid‐phase separation of linear and branched alkane isomers with low molecular weight (<100 g mol^−1^), enriching hexane to 63.35 mole% from an equimolar mixture of isomers. The pressure‐driven process enabled by CMP membranes was shown to be several orders of magnitude faster in transporting linear hexane compared to state‐of‐the‐art membranes and membrane processes. Such CMP membranes present opportunities for studying structure–transport relationship for both solutes and solvents in confined systems, an area that need to be further explored. The diverse range of pore sizes available in CMPs suggests significant potential for achieving size and potentially shape selectivity, broadening selective separations in diverse industrial applications such as refinement plants, polymer industry and fine chemicals, involving sorting of linear polymers and liquid‐phase separation of low‐molecular‐weight alkane isomers using highly permeable membranes.

## 4 Experimental Section

### Chemicals and Materials

Single‐walled carbon nanotubes (SWCNTs, median length: 1 µm, (6,5) chirality, 0.78 nm average diameter, ≥95%, Sigma‐Aldrich), sodium dodecyl sulfate (SDS, ≥98%, Sigma‐Aldrich), tetrabutylammonium perchlorate (Bu_4_NClO_4_ electrochemical analysis, ≥99.0%, Sigma‐Aldrich), DCM (99.6%, Fisher Scientific), propylene carbonate (PC, HPLC, 99.7%, Sigma‐Aldrich), ACN (HPLC, ≥99.9%, Sigma‐Aldrich), carbazole (≥95%, Sigma‐Aldrich), TCB (97%, Sigma‐Aldrich), TCTA (97%, Sigma‐Aldrich), oxytetracycline dihydrate (MW = 496.46 g mol^−1^, ≥95%, Sigma‐Aldrich), glycerol ethoxylate (MW ≈ 1000 g mol^−1^, Sigma‐Aldrich), vitamin B_12_ (VB12, MW = 1355.37 g mol^−1^, ≥98%, Sigma‐Aldrich), PEG 1000, 2000, 4000 (Sigma‐Aldrich), potassium iodide (≥99%, Sigma‐Aldrich), iodine (99.8%, Ajax Finechem.), barium chloride dihydrate (≥99%, Alfa Aesar), ethanol (99.8%, VWR Chemicals), methanol (99.8%, Fisher Scientific), acetone (99%, Aik Moh Paints & Chemicals PTE Ltd.), hexane (≥98.5%, Fisher Scientific), heptane (99%, Sigma‐Aldrich), decane (≥95%, Sigma‐Aldrich), 2‐methylpentane (2,2‐MP, >98%, Tokyo Chemical Industry Co., Ltd.), 2,3‐dimethylbutane (2,2‐DMB, >98%, Tokyo Chemical Industry Co., Ltd.), 2,2‐Dimethylbutane (2,2‐DMB, >98%, Tokyo Chemical Industry Co., Ltd.), and cyclohexane (≥99%, Fisher Scientific). Commercial polypropylene (PP) membrane filters (pore size: 0.1 µm) were purchased from Sterlitech. ITO glass was supplied by South China Xiangcheng Technology Co., Ltd. All chemicals were used without further purification unless otherwise specified.

### Characterization Methods

SEM images of the membranes were taken by FESEM (JEOL JSM‐7610F) with a constant accelerating voltage of 5 kV. For cross‐sectional imaging, the membranes were cryofractured in liquid N_2_. The membranes were sputtered with platinum to achieve proper conductivity. The chemical structure of the membranes and monomers were analyzed by FTIR spectroscopy (Bruker Tesnor 27 FTIR). CMP membranes were grown on the ITO glass by electropolymerization and peeled from ITO glass for FTIR characterization. Goniometer (VCA Optima, AST Products Inc.) was applied to measure the water contact angle (WCA) of the membranes. Before testing, the membranes were dried overnight at 60 °C under vacuum. Atomic force microscope (AFM) image presenting the peak force quantitative nanomechanical mapping (PFQNM) and the corresponding Young's modulus profile of the PTCB membrane were obtained from Bruker Dimension Icon Microscopy. To obtain sufficient membrane samples for nitrogen adsorption measurement, CMP membranes were grown on the ITO glass by electropolymerization and then washed by DCM to remove supporting electrolyte, unreacted monomers, and oligomers. The membranes were peeled from ITO glass and then dried at 60 °C under vacuum. Nitrogen‐physisorption experiments were conducted at 77 K via a surface characterization analyzer (Micromeritics ASAP 2460). Prior to measurement, the samples were degassed at 395 K for 24 h. Low‐field nuclear magnetic resonance (LF‐NMR) relaxation measurements were performed with a 12 MHz NMR analyzer (MesoMR12‐060V‐I). The T_2_ relaxation time was determined using the Carr–Purcell–Meiboom–Gill (CPMG) sequence.

### Fabrication of CNT/PP Membranes

The CNT/PP membranes were fabricated according to previously reported method.^[^ ^12a^ ^]^ SWCNTs dispersion was prepared via a probe sonicator (Fisher Scientific FB705). 8 mg SWCNTs and 400 mg SDS were added into 200 mL deionized (DI) water and were then ultrasonicated for 4 h under 40 W, followed by centrifugation at 10 000 rpm for 1 h (Eppendorf, Centrifuge 5810R) to remove the undispersed SWCNTs. 10 mL of the collected SWCNTs supernatant was filtered through the commercial PP membrane filter to form CNT/PP supporting membranes with the effective diameter of 35 mm. After washing away the SDS on the surface by DI water, the CNT/PP supporting membrane was then dried overnight at 60 °C under vacuum.

### Fabrication of CMP Membranes

The electropolymerization was performed in a three‐electrode cell attached to electrochemical workstation (AUTOLAB, PGSTAT302N). CV was performed at the scanning rate of 50 mV s^−1^. CNT/PP supporting membrane, titanium metal plate, and Ag/AgCl electrode were used as the working, counter, and reference electrodes, respectively. The electrolyte solutions included monomer and 0.1 m Bu_4_NClO_4_ in a mixture of DCM, PC, and ACN (5/3.2/1.8, *v*/*v*/*v*). Before electropolymerization, the electrolyte solutions were degassed with N_2_ for 0.5 h. After electropolymerization, the as‐synthesized membranes were washed by a mixture of DCM and ACN to remove supporting electrolyte, unreacted monomers, and oligomers. Finally, the membranes were dried in a vacuum oven at 60 °C for 12 h.

### Permeation Experiments

Separation performance was performed in a homemade dead‐end stirred cell at 6 bar under room temperature (≈22 °C) in air‐conditioned environment unless otherwise specified. A stainless‐steel reducer with a 0.5 cm hole at the center was used to seal the membrane to obtain active surface of around 0.196 cm^2^. The cross‐flow performance of the membrane was evaluated via a lab‐built cross‐flow setup. The effective membrane area in the cell was 0.196 cm^2^. The applied pressure and cross‐flow rate were kept at 6 bar and 300 mL s^−1^ respectively during the measurements. The permeates were collected after the system achieved stable state. The permeance (*J*, L m^−2^ h^−1^ bar^−1^) was calculated as follows

(3) $$J=\frac{V}{A\;\times \;\Delta t\;\times \;\Delta P}$$

where *V* (L) is the volume of permeate, *A* (m^2^) is the effective area of the membrane, *∆t* (h) is the operating time for collecting permeate, and *∆P* (bar) is the applied transmembrane pressure. The rejection of the membrane was measured using feed solutions containing solutes with various molecular weights and structure (Table S1, Supporting Information). The solute rejection (*R_i_*, %) was calculated as follows

(4) $$R_{i}=\frac{\left(C_{F,\;i}-\;C_{P,\;i}\right)\;\times 100}{C_{F,\;i}}\;$$

where *C*_F_ *_, i_* and *C*_P_ *_, i_* are the concentration of solutes in feed and permeate solutions, respectively. The concentration of PEG and glycerol ethoxylate in feed and permeate solutions was determined via HPLC (for mixed solutes) or spectrophometic method (for single solute) followed previously reported procedure.^[^ ^32^ ^]^ For HPLC, the collected samples were examined using an Agilent Technologies 1260 Infinity II HPLC system, coupled with an evaporative light scattering detector (ELSD, G4260B). The ELSD evaporator temperature was adjusted to a temperature of 30 °C and the nebulizer temperature was set at 25 °C. N_2_ gas was introduced into the detector at a rate of 1.8 SLM. The HPLC pump was operated at a flow rate of 1 mL min^−1^ and the column was maintained at a temperature of 25 °C. The analysis was conducted using a C18 column (4.6 × 150 m) from Agilent Technologies, and the mobile phases consisted of acetonitrile and deionized water. Other solute concentrations were quantified by ultraviolet–visible (UV–vis) spectrometer (Agilent Technologies, Cary 60). The permeate and feed were diluted to get proper UV–vis spectra measurement.

Hexane isomer separation was performed in dead‐end stirred cell at 6 bar under room temperature (≈22 °C). The Gas Chromatography/Mass Selective Detector (Agilent 5975C) was utilized to analyze both the feed and permeate samples. Chromatograph analysis and peak integration were performed using the 5975c MSD Data Analysis software. A temperature ramp with a rate of 5 °C min^−1^, ranging from 45 to 100 °C was used in the GC analysis. The feed mixtures were prepared based on molar ratios. The separation factor (𝛼_1/2_) was calculated as follows

(5) $$\alpha_{1/2}=\frac{x_{1,\;P}}{x_{2,\;P}}/\frac{x_{1,\;F}}{x_{2,\;F}}$$

where *x*_1, *P*_, *x*_2, *P*_, *x*_1, *F*_, and *x*_2, *F*_ are the mol fraction of species 1 and 2 in permeation solution and feed, respectively.

### Molecular Dynamics Simulations

*Quantum Chemical Calculations*: The monomer, dimer, and trimer of TCTA, and the model structure of PEG1000 and single pore of TCTA membrane were optimized with M06‐2X method by the Gaussian 09 package^[^ ^33^ ^]^ with a compound basis set; the 6‐311+G(d) basis set was used for all bridgehead C and N atoms and the 6‐31G(d) basis set was employed for the H and non‐bridgehead C atoms.

*Construction of Atomic Models*: The rejection of TCTA membrane to three solutes (PEG1000, OTC, and VB_12_) in ethanol was simulated in a system as shown in Figure S21 (Supporting Information). Two chambers with feed and permeate sides were separated by the TCTA membrane with the thickness of four layers. The graphene plates acted as the pistons on the left and right sides of the chambers could freely move on the *Z*‐axis. The MD simulation was conducted at 300 K with *p*_left_ = 501 bar and *p*_right_ = 1 bar, giving pressure difference of 500 bar to decrease thermal noise and increase the signal/noise ratio within the simulation time scale of a nanosecond.^[^ ^34^ ^]^ 10 PEG chains with 10 units, 10 OTC molecules, and 4 VB_12_ molecules were added into the ethanol with 20 000 molecules at the feed side, respectively, to achieve solute concentration of 5000 ppm, which was around one order of magnitude higher than the experimental filtration process to enhance the calculation accuracy. First, a 40‐cycle annealing process was carried out, and a 2.0 ns MD simulation at 500 K was performed in the constant‐temperature, constant‐volume (*NVT*) ensemble with the Condensed‐phase Optimized Molecular Potential for Simulation Studies (COMPASS) force field. Then, the temperature was gradually reduced to 300 K within 2.0 ns, and the simulation system was relaxed by constant‐temperature, constant‐pressure (*NPT*) simulations (300 K and 1.0 atm). Finally, to further relax the membrane, a 10 ns MD simulation was performed at 300 K to realize a final stable membrane. This structure was used to construct a histogram of N···N distances between two adjacent TCTA moieties. Along the trajectory, membrane structures calculated after every 300 ps were extracted and 10 structures were selected, and a histogram was built. According to the histogram, the pore size was determined by the probability of N1···C1 distances. The energy minimization was done by the steepest descent method with a maximum step of 0.5 Å and a force tolerance of 1000 kJ/(mol nm), and velocities were assigned according to the Maxwell–Boltzmann distribution at 300 K. The electrostatic interactions were calculated by the particle‐mesh Ewald method. The time step was 2 fs and the trajectory was saved every 50 ps. In addition, to gain a better understanding of the conformation change in linear PEG chains as they diffuse through PTCTA membrane, this study opted for a thicker TCTA membrane with a width of 3.8 nm, replacing the previously used thin TCTA layers in the penetration process model. Specifically, 10 PEG chains with 10 units were also added into the ethanol at the feed side and two graphene plates acting as the pistons can freely move on the Z‐axis. Periodic boundary conditions were applied solely in *x*‐ and *y*‐directions. All the simulations were conducted using Gromacs v.5.0.6,^[^ ^35^ ^]^ and Materials Studio (MS) programs.

## Conflict of Interest

The authors declare no conflict of interest.

## Supporting information



Supporting Information

## Data Availability

The data that support the findings of this study are available from the corresponding author upon reasonable request.
