# Supplementary material for: Shape‐Selective Molecular Separations Enabled by Rigid and Interconnected Confinements Engineered in Conjugated Microporous Polymer Membranes
Source: Adv Sci (Weinh). 2025 Apr 17;12(22):2416266. doi: 10.1002/advs.202416266 (PMC12165078; doi:10.1002/advs.202416266)
Supplement: Supplementary file 1 — Supporting Information [file ADVS-12-2416266-s001.pdf]

ADVANCED SCIENCE
Open Access

Supporting Information

for *Adv. Sci.*, DOI 10.1002/advs.202416266

Shape-Selective Molecular Separations Enabled by Rigid and Interconnected Confinements Engineered in Conjugated Microporous Polymer Membranes

Yanqiu Lu, Hao Deng, Liling Zhang, Yong Wang and Sui Zhang**

Supporting Information for

Shape-Selective Molecular Separations Enabled by Rigid and Interconnected Confinements Engineered in Conjugated Microporous Polymer Membranes

Yanqiu Lu,[1,2,3†] Hao Deng,[1†] Liling Zhang,[4] Yong Wang,[2] Sui Zhang[1,3*]*

Supporting Information Text

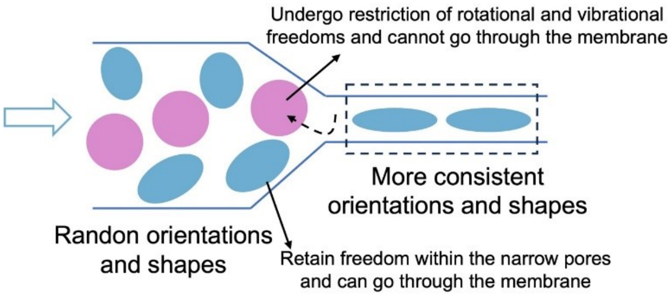

Figure S1 Schematic illustration of restriction of rotational and vibrational freedoms of the molecules with different shapes in the confined channel.

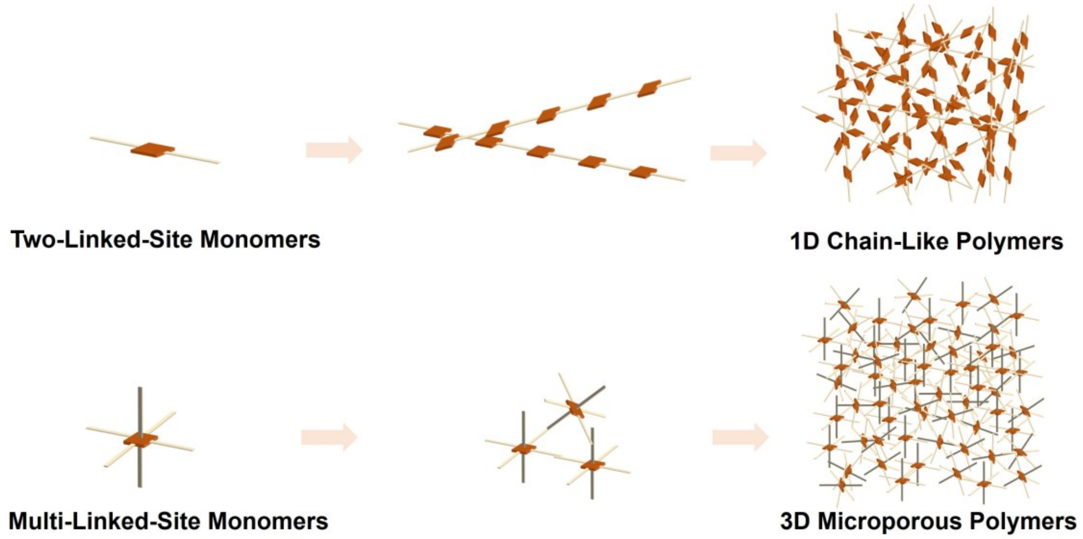

Figure S2 Schematic representation of 1D chain-like CMPs and 3D CMPs composed by two-linked-site monomers and multi-linked-site monomers.

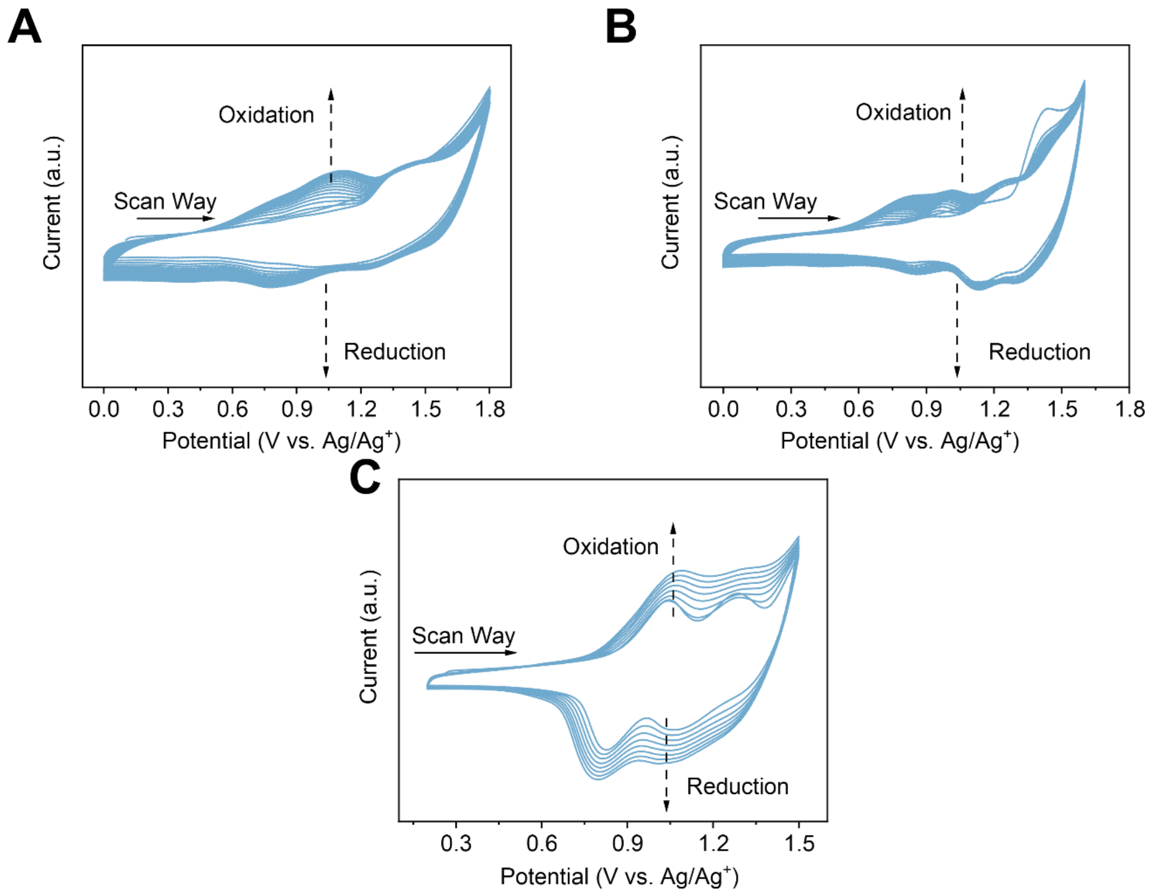

Figure S3 The multicycled CV curve of (a) PCz, (b) PTCB and (c) PTCTA membrane.

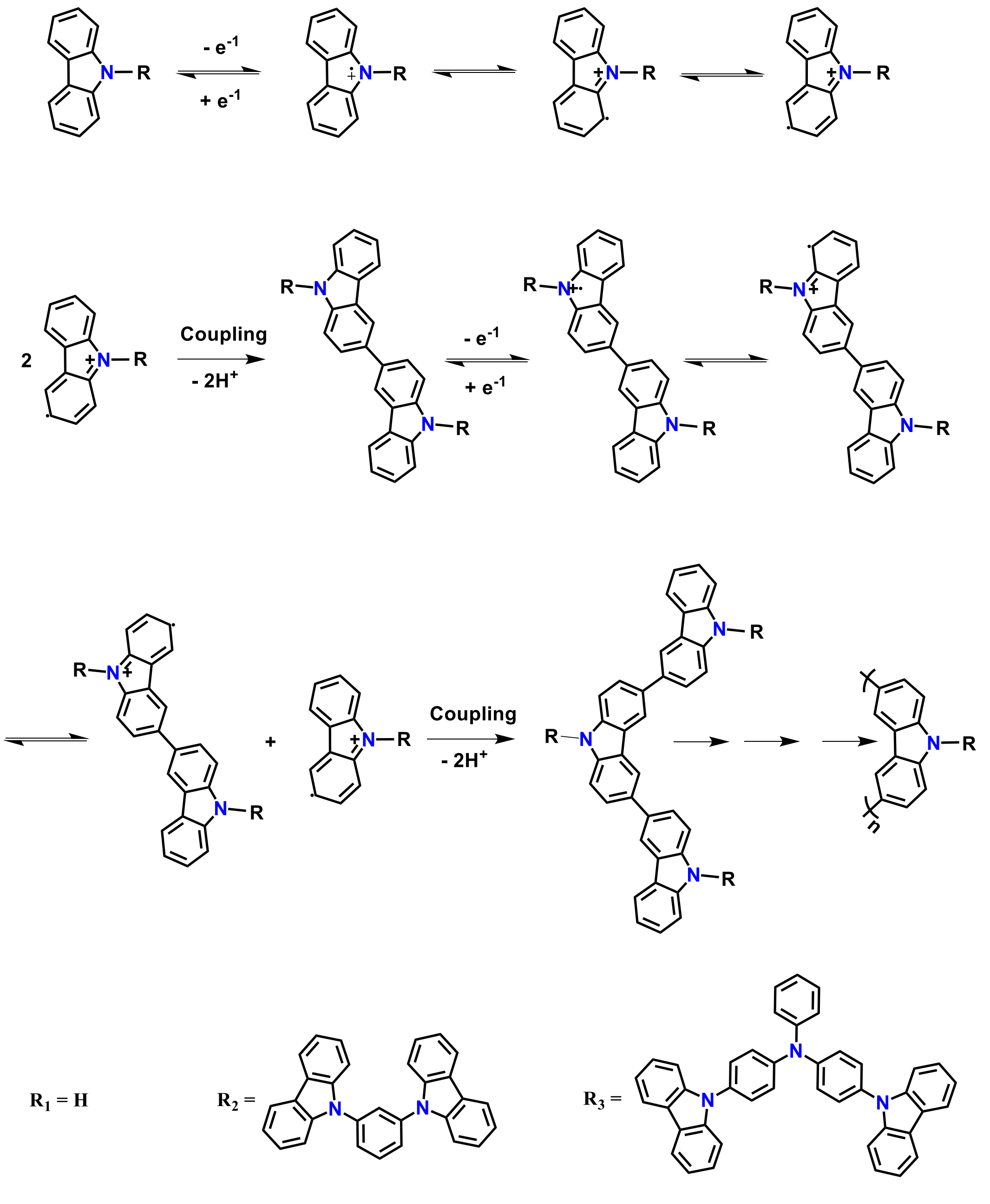

Figure S4 The electropolymerization mechanism of Cz (R_1), TCB (R_2), and TCTA (R_3).

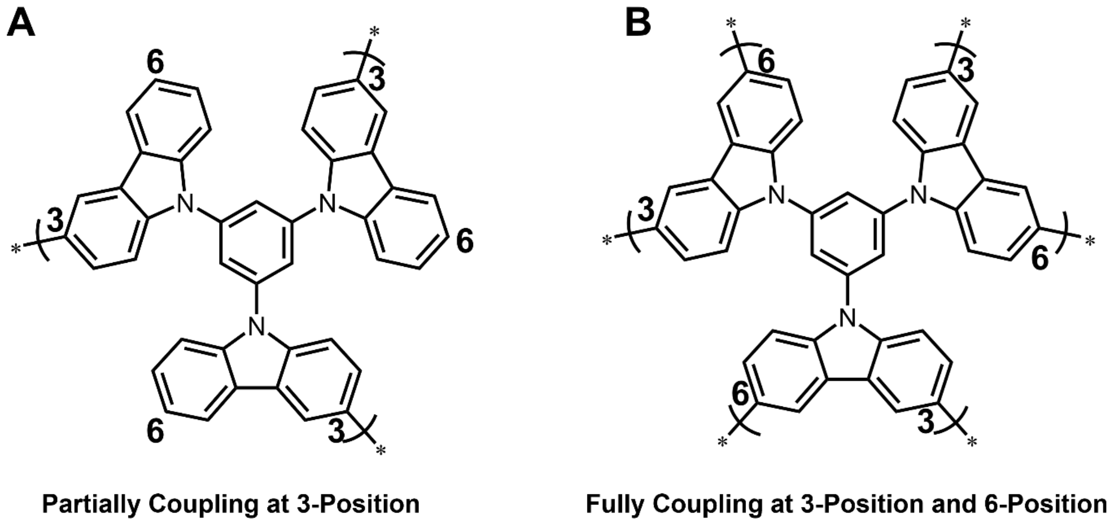

Figure S5 A schematic illustrating TCB monomers (A) partially coupling at 3-position of the carbazole groups and (B) fully coupling at 3-position and 6-position of the carbazole groups.

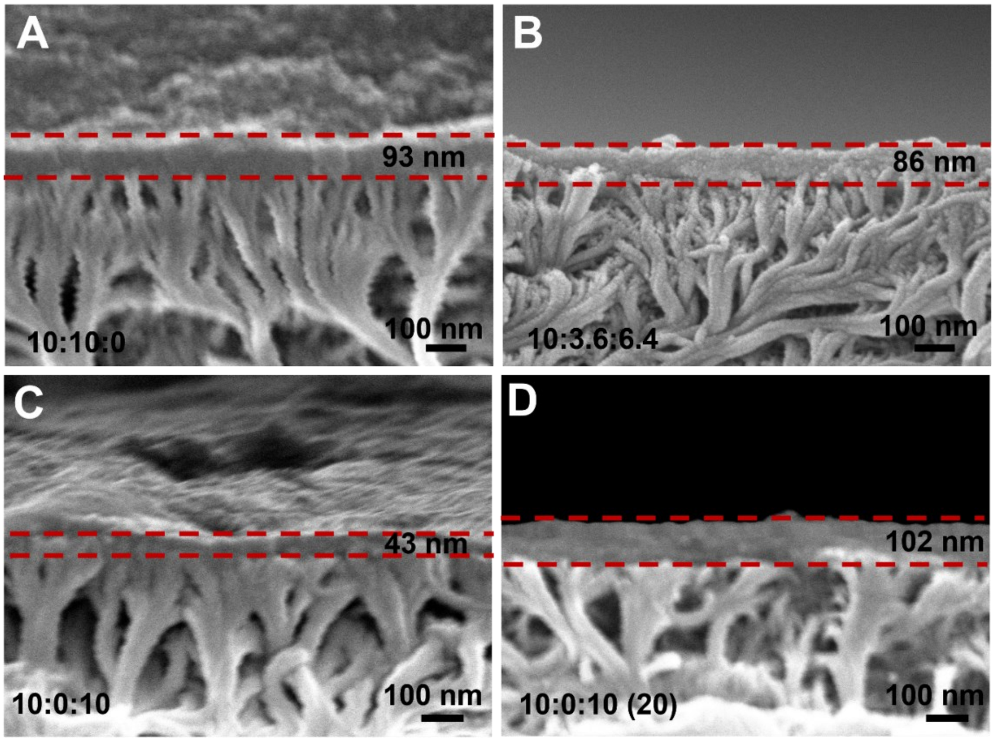

Figure S6 SEM images of the cross section of PTCB membranes electropolymerized in three different electrolyte solutions.

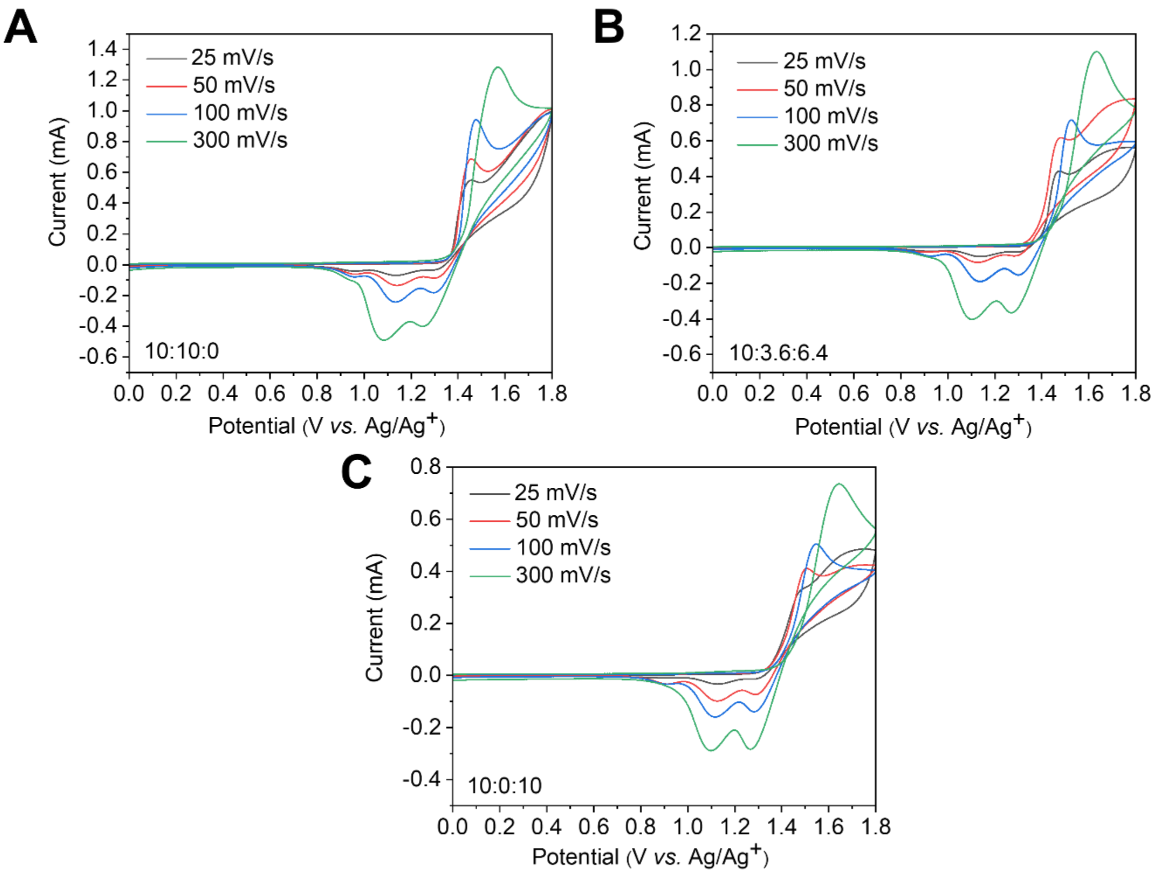

Figure S7 The first CV curve of TCB electropolymerized in three different electrolyte solutions on ITO glass under different scan rates.

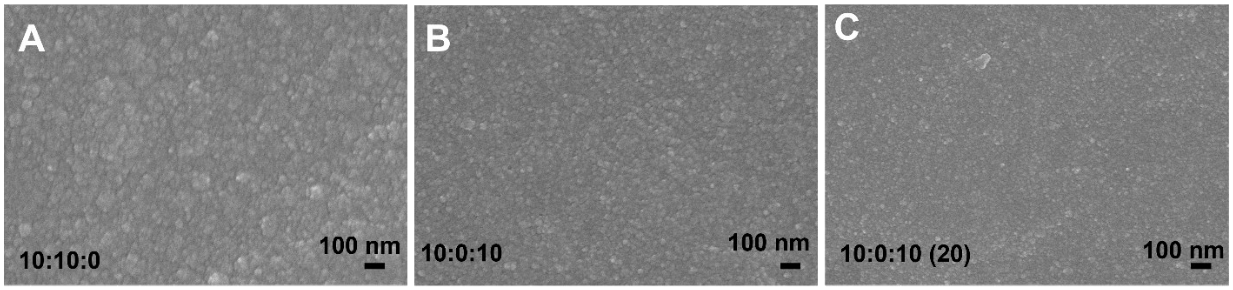

Figure S8 SEM images of PTCB membranes electropolymerized in electrolyte solution with (A) DCM: ACN: PC ratio of 10: 3.6: 6.4 (*v*/*v*/*v*), (B) DCM: ACN: PC ratio of 10: 0: 10 (*v*/*v*/*v*), (C) DCM: ACN: PC ratio of 10: 0: 10 (v/v/v), with the scan cycles increased to 20.

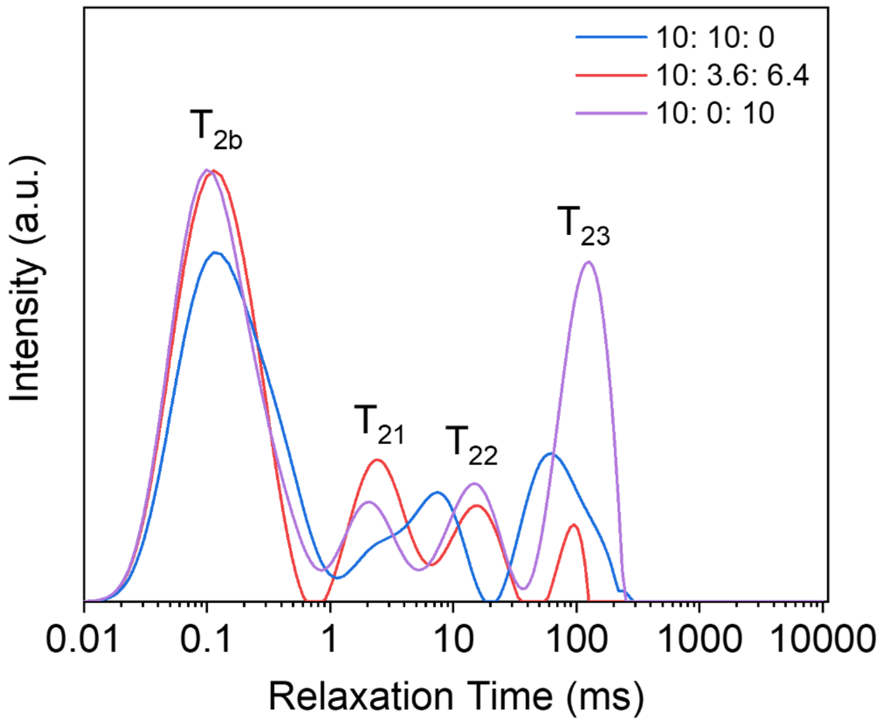

Figure S9 Proton transverse relaxation time T_2 distributions of methanol in PTCB membranes under different electropolymerization conditions.

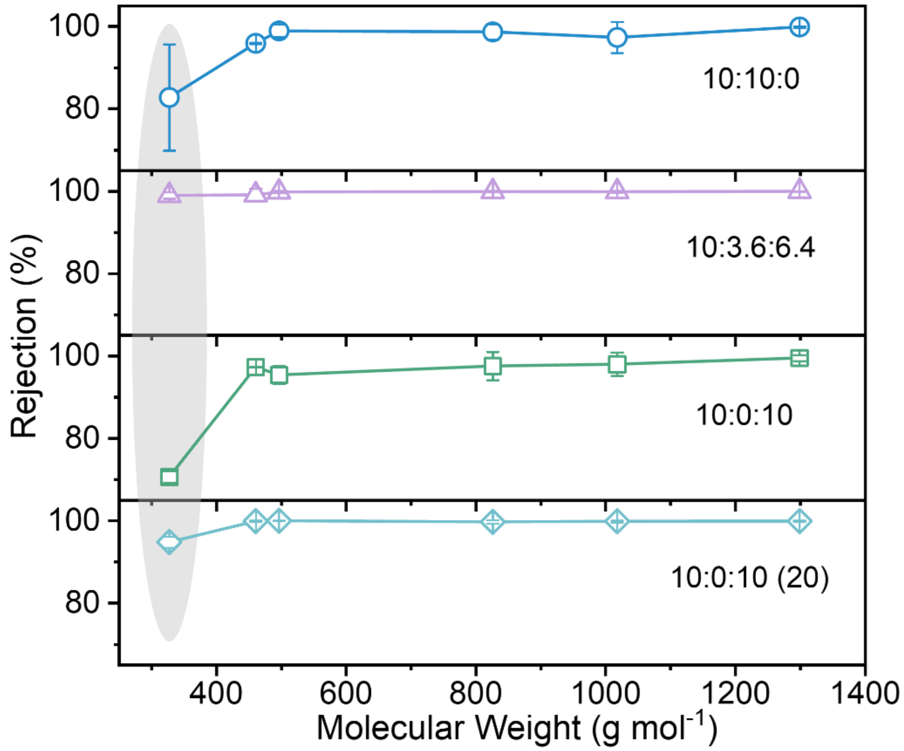

Figure S10 Rejection performance of PTCB membranes electropolymerized in three different electrolyte solutions by 8 scan cycles. Data labelled as "(20)" indicate performance with the scan cycles increased to 20. Solutes include Methyl Orange (MO), Oxytetracycline (OTC), Allura Red AC (ARAC), Brilliant Blue R (BBR), Rose Bengal (RB), Vitamin B_{12} (VB12). The feed solution contains 50 ppm solute in methanol.

Text I: Optimal electropolymerization conditions.

The optimal experimental conditions were explored by changing the electrochemical conditions including the monomer concentrations, oxidation potential ranges, and scan cycles. After optimizing, the monomer concentrations in electrolyte solution are 0.8 mM carbazole for PCz membrane, 0.4 mM TCB for PTCB membrane, and 0.4 mM TCTA for PTCTA membrane. The optimal CV oxidation potential ranges and scan cycles are 0-1.8 V, 20 cycles for PCz membrane, 0-1.8 V, 8 cycles for PTCB membrane, and 0.2-1.5 V, 8 cycles for PTCTA membrane.

As the polymerization proceeded, continuous CMP films with different morphologies were formed on the CNT/PP support as observed in the SEM images (**Figure 1E** and **Figure S11**). The molecular structure of the monomer plays a key role in membrane morphology as well as surface hydrophobicity. It is observed that PCz and PTCB membranes present smoother surface morphology compared to that of PTCTA membrane. The water contact angle (WCA) of the PTCTA membrane was 117.7 ± 4.3° (**Figure S12**), indicating its hydrophobic nature compared to that of CNT/PP support (77.75 ± 1.78°). On the other hand, PCz membrane presented WCA of 66.3 ± 7.9°, thus it is considered to be hydrophilic. The WCA of PTCB membrane was 80.5 ± 1.7°, which was between that of PCz and PTCTA membranes. The variation in hydrophobicity among these three CMPs membranes may be attributed to their distinct membrane structures constructed by different monomers. The chemical structure of the CMP membrane was characterized by FTIR (**Figure 1D and Figure S13**). FTIR spectra of Cz, TCB and TCTA monomer present two vibratory peaks at ~720 and ~748 cm^{-1}, which are assigned to the 1,2-bisubstituted benzene ring in carbazole [1]. Polymerization of Cz, TCB or TCTA monomer was confirmed by the dramatically weakened signal of 1,2- bisubstituted benzene ring and a newly generated characteristic peak of trisubstituted benzene ring at about 803 cm^{-1} [1].

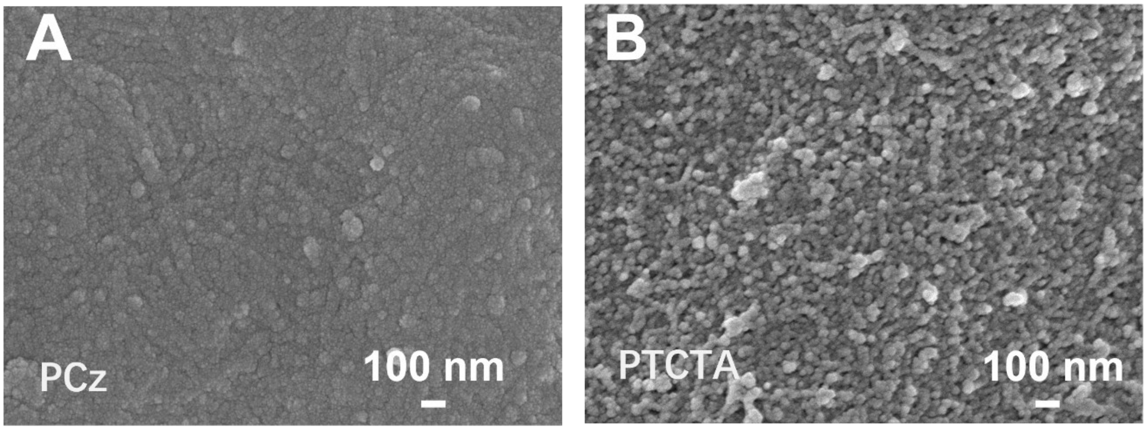

Figure S11 SEM images of (A) PCz films and (B) PTCTA films.

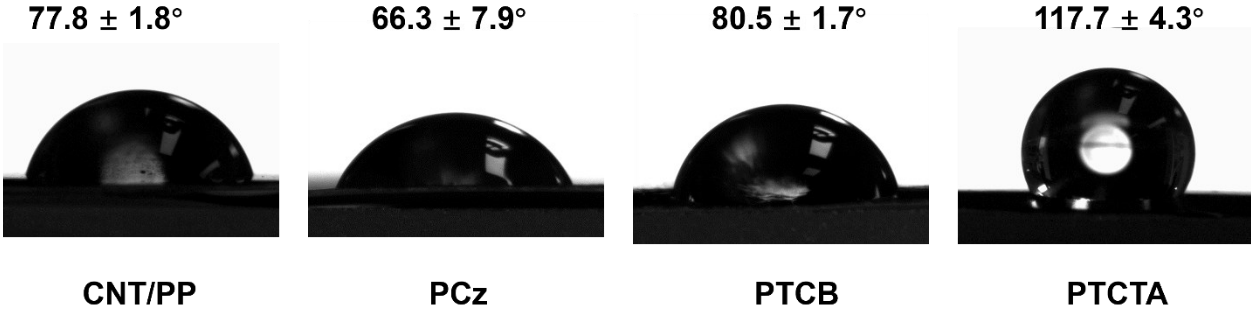

Figure S12 Water contact angles of CNT/PP support, PCz, PTCB and PTCTA membrane.

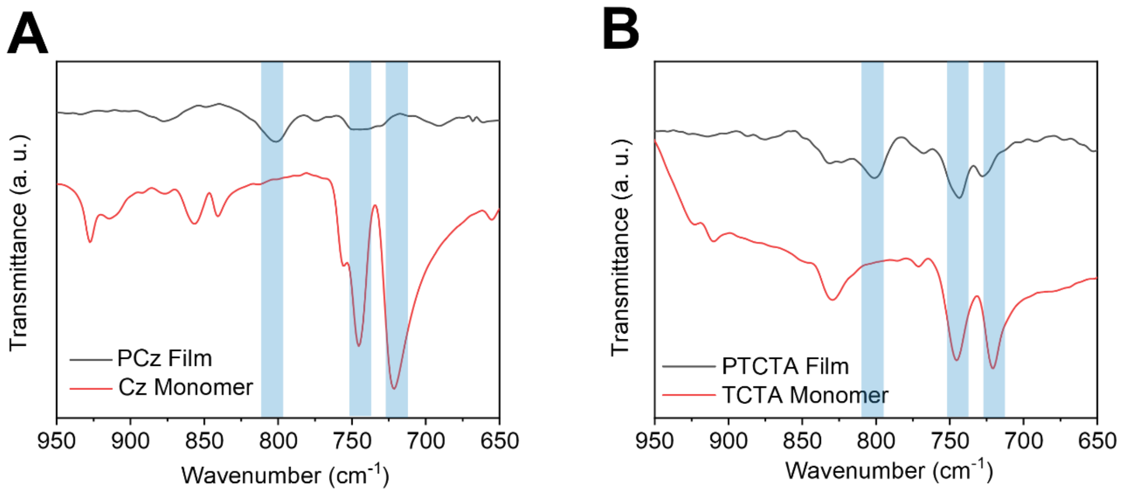

Figure S13 FTIR spectra of (A) PCz membrane and Cz monomer (B) PTCTA membrane and TCTA monomer.

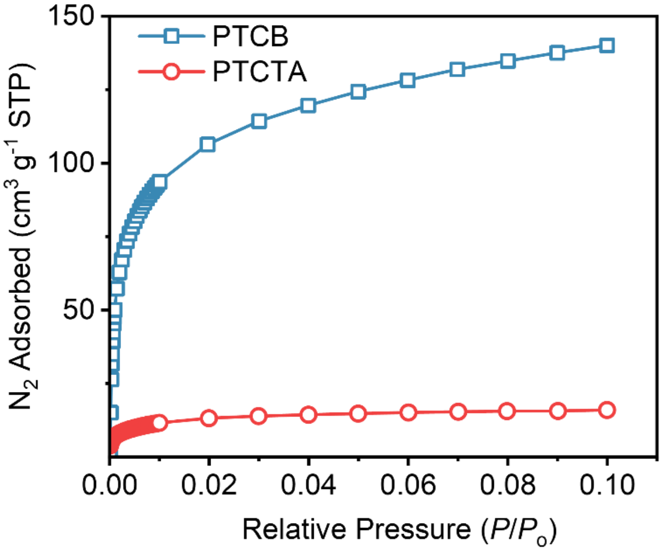

Figure S14 N_2 uptake of CMP membranes. Notably, PTCTA membrane exhibits lower N_2 uptake compared to that of PTCB membrane. The presence of easily oxidizable 1,4-N-substituted phenylene units (as shown in **Figure 1A**) in the TCTA monomer structure can lead to side reactions, resulting in a significant reduction in the microporosity of TCTA-EP membrane [2].

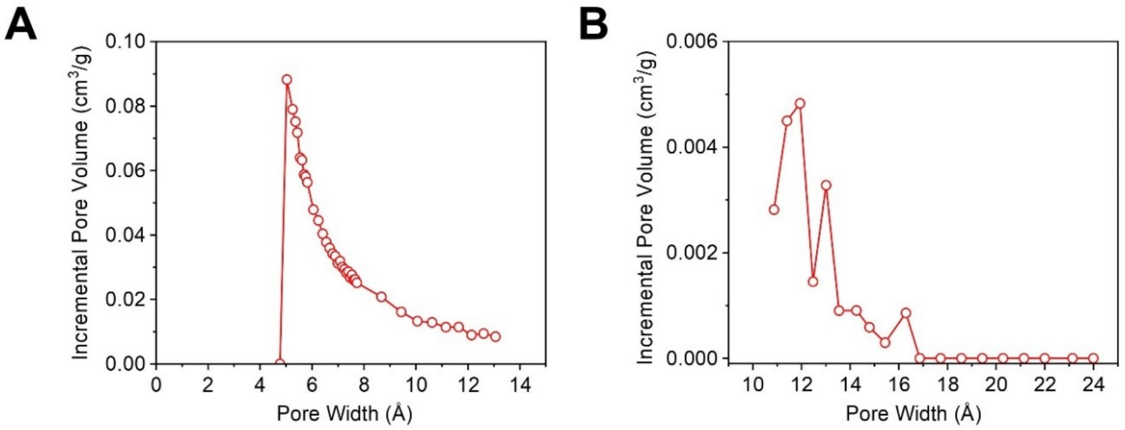

Figure S15 Pore-size distribution profiles of (A) PTCB and (B) PTCTA membranes.

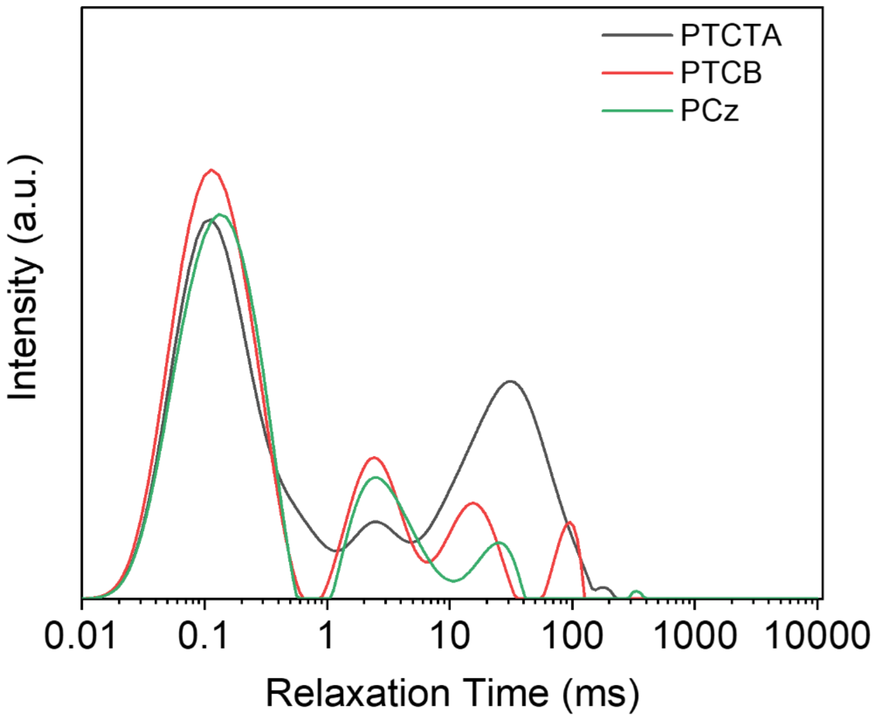

Figure S16 Proton transverse relaxation time T_2 distributions of methanol in CMP membranes fabricated by different monomers.

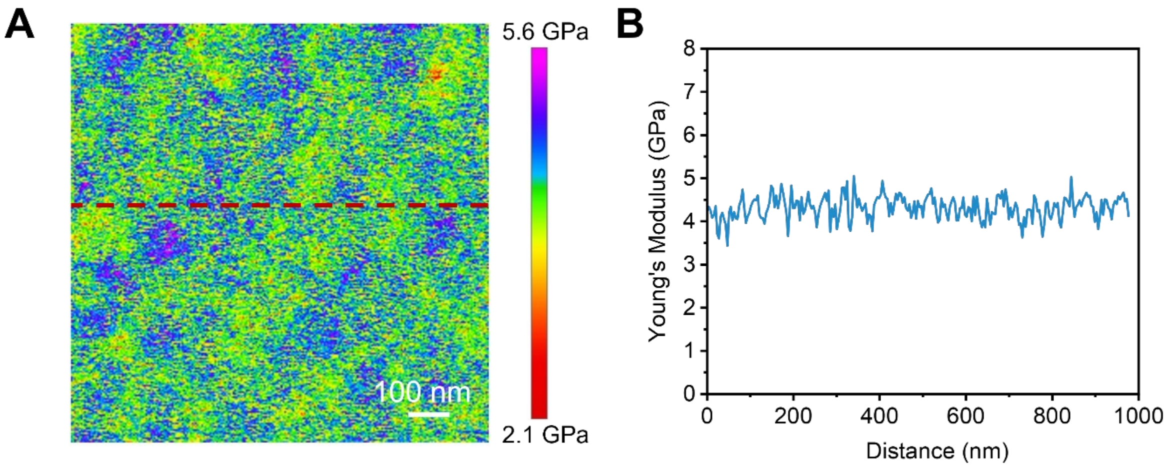

Figure S17 (A) AFM image presenting the peak force quantitative nanomechanical mapping (PFQNM) and (B) the corresponding Young's modulus profile of the PTCB-EP membrane fabricated on ITO.

Table S1 Solute molecules used in this work.

Solute	Chemical Formula	MW (g mol^{-1})	Dimensions (Å^3) ‡	Net Charge
Methyl Orange (MO)	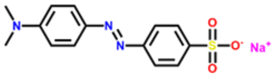	327.33	7.18 × 17.23 × 5.28	-1
Oxytetracycline (OTC)	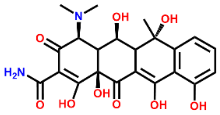	460.46	9.33 × 9.47 × 15.16	0
Allura Red AC (ARAC)	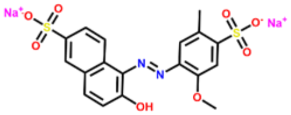	496.42	10.41 × 5.54 × 17.88	-2
Vinyl POSS	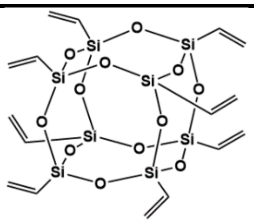	632.31	10.94 × 12.53 × 13.81	0
Brilliant Blue R (BBR)	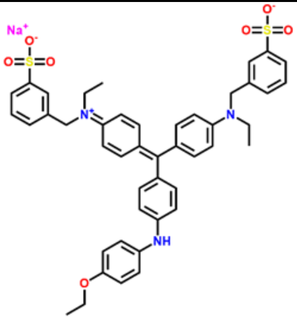	825.97	20.55 × 20.07 × 10.13	-1
PEG1000	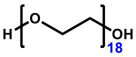	~1000.00	4.22 × 4.64 × 81.93	0
Glycerol Ethoxylate (GE)	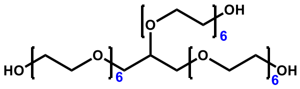	~1000.00	7.62 × 8.87 × 50.29	0

Rose Bengal (RB)	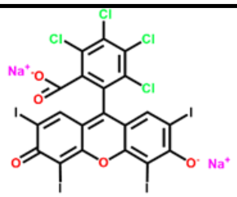	1017.65	9.91 × 14.21 × 14.86	-2
Vitamin B_{12} (VB12)	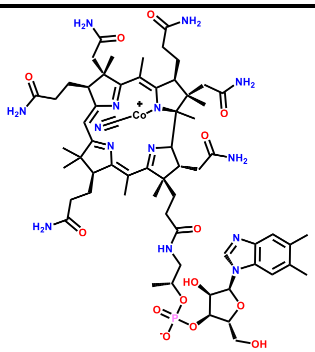	1355.37	12.27 × 16.42 × 23.02	0

‡The three-dimensional size of the solutes was estimated as illustrated according to our previous method [3].

Table S2 Summary of the permeance for PCz, PTCTA, PTCTA membranes in this work.

Membrane	Solvent/Solute	Permeance (L m^{-2} h^{-1} bar^{-1}) /Rejection (%)
PCz	Ethanol	0.90
	Methanol	5.89
	Tetrahydrofuran	1.93
	Acetonitrile	19.26
	Acetone	10.86
	Hexane	0
	Ethanol/500 ppm PEG 1000	0.11/80.04
	Ethanol/500 ppm OTC	0.60/99.02
	Ethanol/500 ppm VB12	0.42/99.62
PTCTA	Ethanol	15.98
	Methanol	44.73
	Tetrahydrofuran	39.12
	Acetonitrile	45.46
	Acetone	55.77
	Hexane	60.10
	Ethanol/500 ppm PEG 1000	4.40/9.83
	Ethanol/500 ppm OTC	2.45/77.53
	Ethanol/500 ppm VB12	6.43/96.44
PTCB	Ethanol	6.12
	Methanol	10.73

	Tetrahydrofuran	6.38
	Acetonitrile	24.13
	Acetone	21.74
	hexane	5.73
	Heptane	4.80
	Decane	1.93
	2-methylpentane	0.69
	cyclic cyclohexane	0.11
	2,3-dimethylbutane	0.04
	2,2-dimethylbutane	0.10
	Ethanol/500 ppm PEG 1000	2.25/15.60
	Ethanol/500 ppm OTC	1.97/99.08
	Ethanol/500 ppm VB12	4.61/99.43

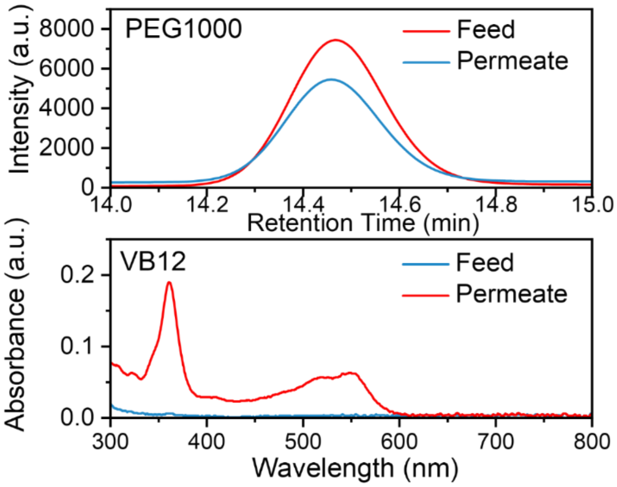

Figure S18 Gel permeation chromatography (GPC) curve and ultraviolet-visible (UV-vis) absorbance curve of the feed including PEG1000 and VB12 and permeate after filtration through PTCB membrane by cross-flow mode. The permeate and feed were diluted to get proper GPC and UV-vis spectra measurement. The rejections to PEG100 and VB12 are 29.94% and 99.47%, respectively.

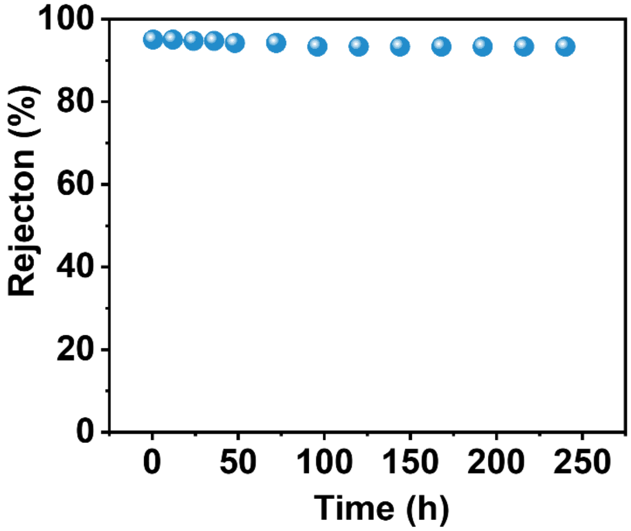

Figure S19 Long-term rejection of PTCB membrane to VB12 in cross-flow mode under a flow rate of 300 ml s^{-1}.

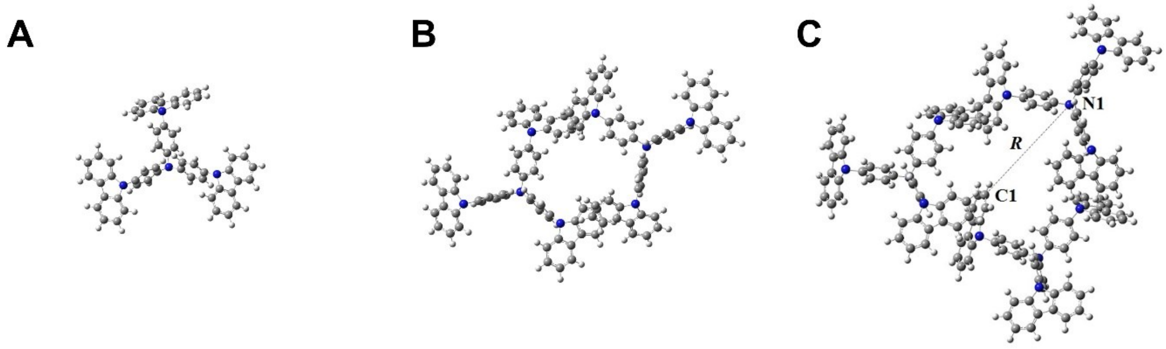

Figure S20 TCTA monomer and dimer and trimer.

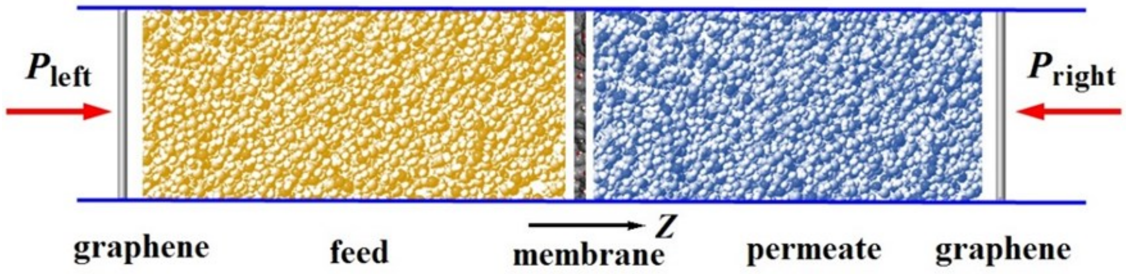

Figure S21 A simulation system for molecular permeation.

<u>**Text II: Pore-flow model of PTCTA membrane.**</u>

The correlations between solvent permeance and Hansen solubility parameters and the diameter of the solvents are nonlinear (R^2 lower than 0.1) as shown in **Figure S22A** and **B**. Additionally, the product of permeance and viscosity is constant, independent of Hansen solubility parameters (**Figure S22C**), indicating that these factors might not significantly affect solvent transport. All these phenomena suggest that solvent transport in TCTA-EP membranes follows the pore-flow model, as supported by previous studies [4].

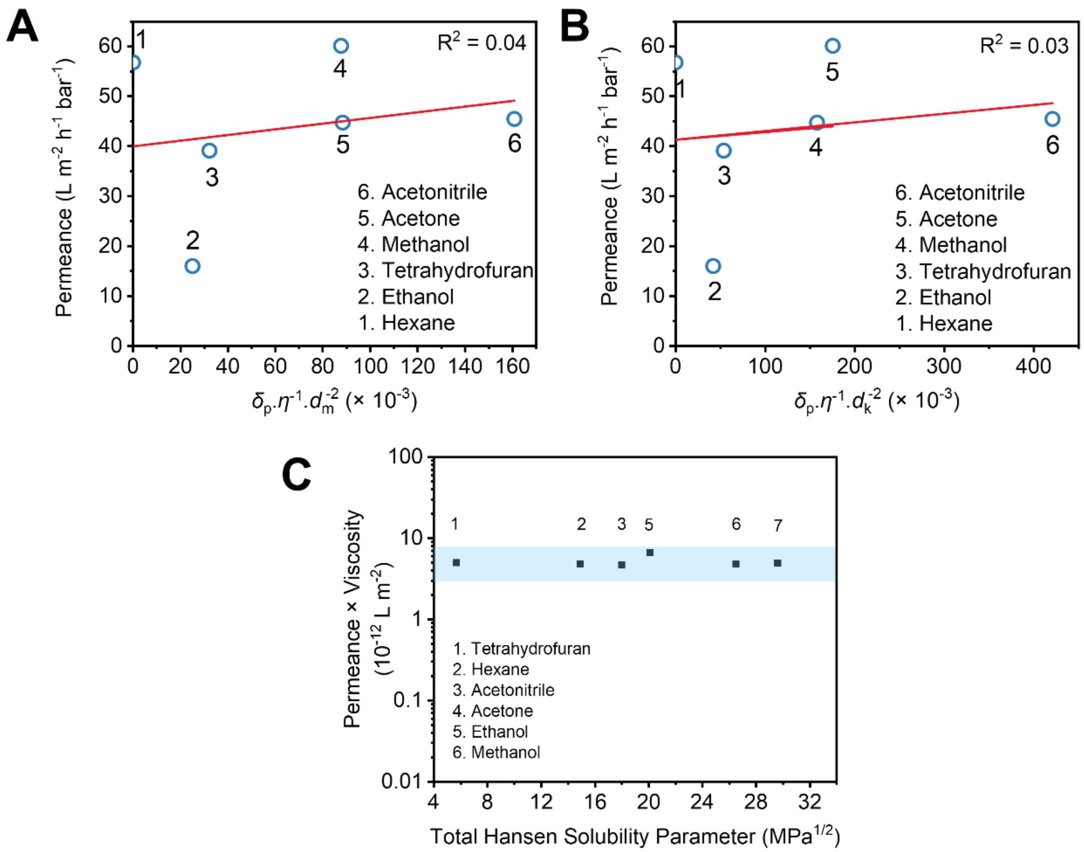

Figure S22 The solvent permeance of the PTCTA membrane against (A) solubility parameter multiplied by inverse of viscosity and molar diameter (d_m), (B) solubility parameter multiplied by inverse of viscosity and kinetic diameter (d_k). (C) Product of permeance and viscosity of different solvents against total Hansen solubility parameter for PTCTA membrane.

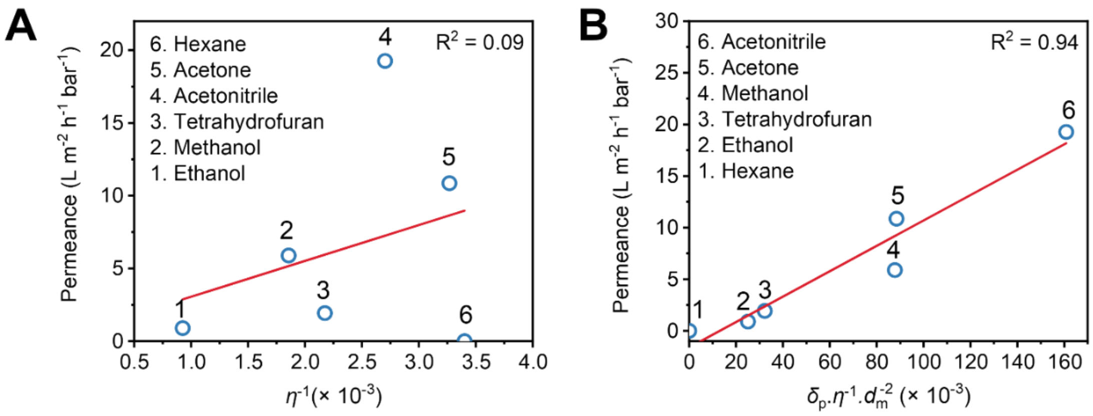

Figure S23 The solvent permeance of the PCz membrane against the (A) inverse of viscosity and (B) solubility parameter multiplied by inverse of viscosity and molar diameter.

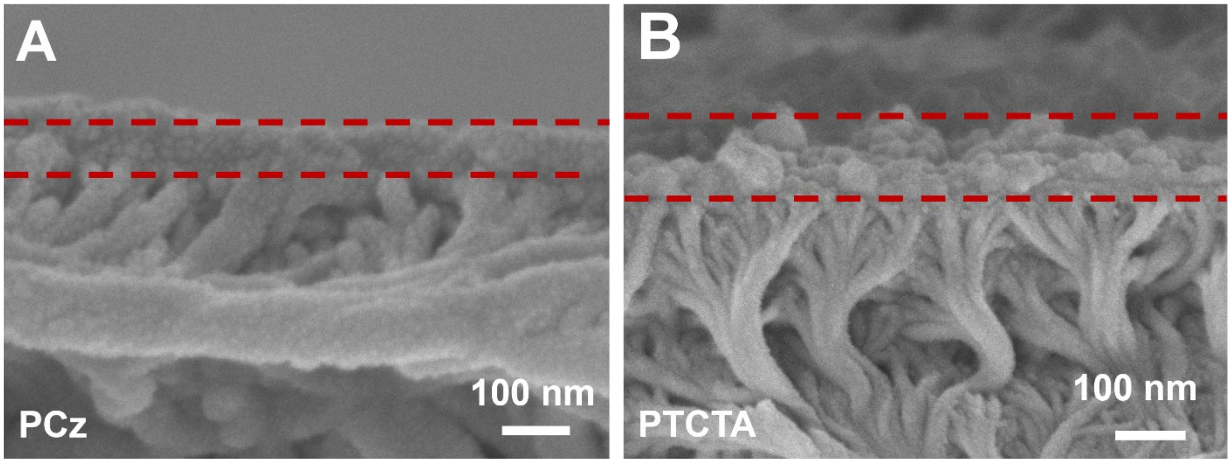

Figure S24 SEM images present the cross section of PCz and PTCTA membrane.

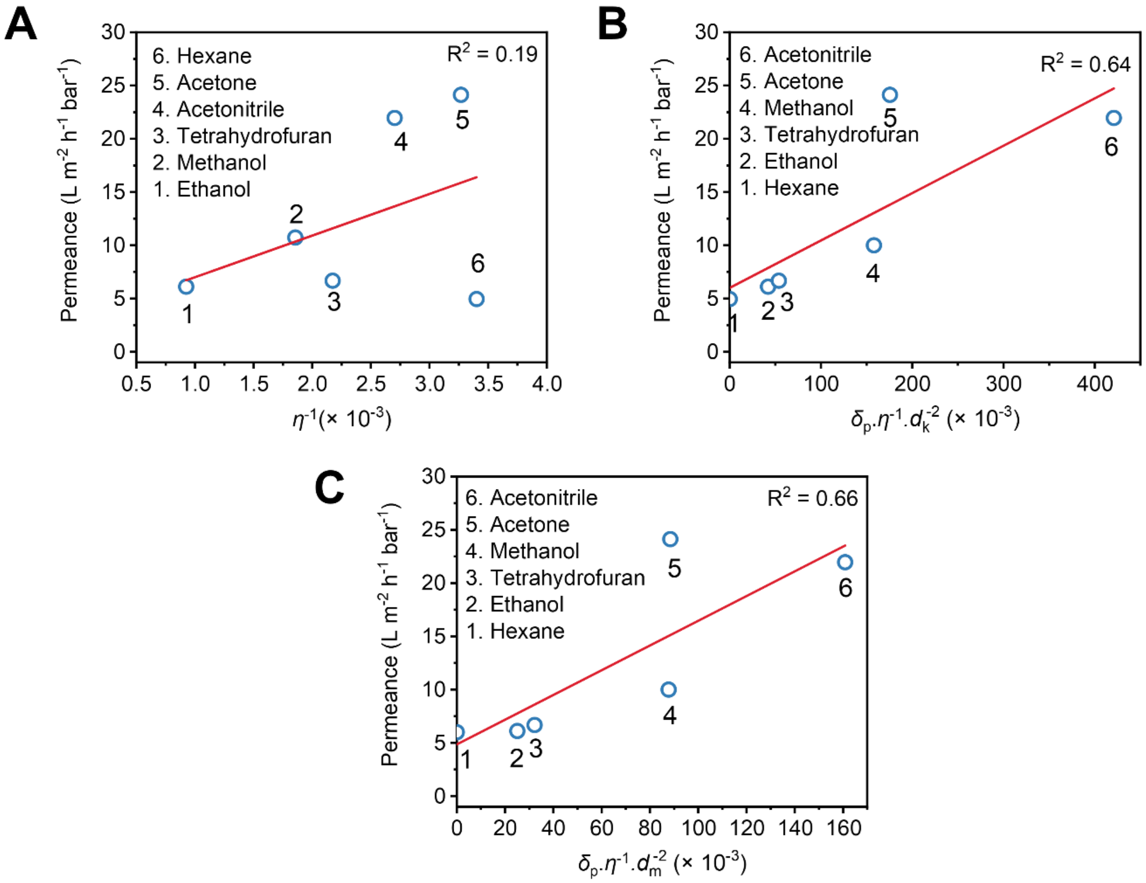

Figure S25 (A) The solvent permeance of the PTCB membrane against the inverse of viscosity, (B) solubility parameter multiplied by inverse of viscosity and kinetic diameter (d_k), (C) solubility parameter multiplied by inverse of viscosity and molar diameter (d_m).

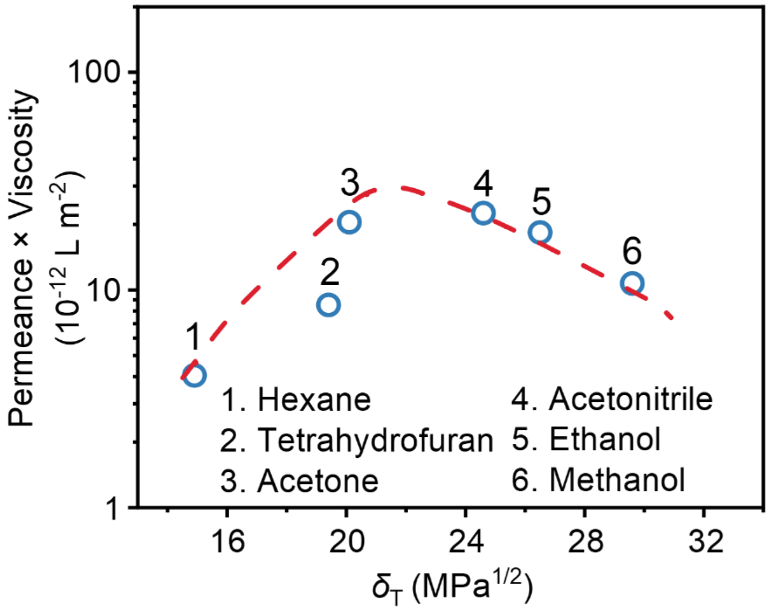

Figure S26 Product of permeance and viscosity of different solvents as the function of total HSP (δ_T) of the solvent for PTCB membrane.

Table S3 The properties of the solvents used in this work.

Solvents	Viscosity at 25 °C (μ) †	Total Hansen Solubility Parameter (δ_T) ‡	δ_p	Kinetic Diameter (d_k) §	Molar Diameters (d_m) ¶
	mPa·s	$MPa^{1/2}$	$MPa^{1/2}$	nm	nm
Ethanol	1.08	26.5	8.8	0.44	0.57
Acetone	0.31	20.1	10.4	0.44	0.62
Methanol	0.54	29.6	12.3	0.38	0.51
Hexane	0.29	14.9	0.0	0.43	0.75
Heptane	0.30.55	15.3	0.0	0.43	0.78
Decane	0.85	15.7	0.0	0.43	0.85
Acetonitrile	0.37	24.6	18.0	0.34	0.55
Tetrahydrofuran	0.46	19.4	5.7	0.48	0.62
Cyclohexane	0.88	16.8	0.0	0.60	0.70
2-methylpentane	0.27	/	/	0.5	0.75
2,2-dimethylbutane	0.33	/	/	0.62	0.63
2,3-dimethylbutane	0.39	/	/	0.58	0.74
PCz	/	19.72	/	/	/
PTCB	/	20.32	/	/	/
PTCTA	/	20.31	/	/	/

† Viscosity (μ) of solvents was obtained from reference [5];

‡ Total Hansen solubility parameter (δ_T) of solvents was obtained from reference [6];

§ Kinetic diameter (d_k) of solvents was obtained from reference [7];

¶ Molar diameter (d_m) of solvents was calculated from: $d_m = 2 \times (3V_m / 4\pi N_A)^{1/3}$; where N_A is the Avogardo's number [8].

Text III: Selective separation of butanol and acetone.

Acetone-butanol-ethanol (ABE) fermentation is a well-established bioprocess for the production of alternative energy and biobased chemicals [9]. However, traditional separation techniques such as distillation and pervaporation are often hindered by high energy consumption and low separation efficiency. To address this issue, PTCB membranes were employed for the selective separation of mixtures typically found in such processes, such as those containing 40 g/L acetone, 150 g/L butanol, 10 g/L ethanol, with the rest being water [10]. As shown in **Figure S27**, the concentrations of butanol and ethanol decrease relative to the feed, with separation factors of 3.68 for acetone/butanol and 3.52 for acetone/ethanol. These results highlight the potential to leverage the different affinities between the solvent molecules and the PTCB membrane for effective solvent separation.

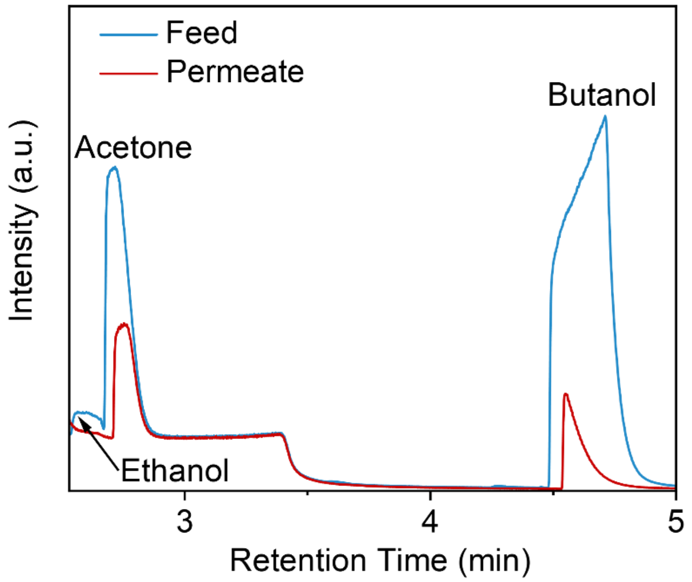

Figure S27 GC spectra of the acetone-butanol-ethanol feed and permeate through PTCB membranes under 6 bar. Headspace gas chromatography-mass spectrometry (HS-GC-MS, Agilent 7890B/5977A) was utilized to analyze both the feed and permeate samples.

Table S4 Comparison of membrane separation factor, hexane flux and operating conditions.

Membrane [Ref.]	Feed composition	Process type	Phase of the feed	Phase of the permeate	Operating temperature [°C]	Hexane flux [mol m^{-2} s^{-1}]	Separation factor (hexane/di-branched isomers)
PTCB (This work)	Hexane/22DMB (1:1 in mol)	Pressure-driven	Liquid	Liquid	25	3.20×10^{-2}	1.65
PTCB (This work)	Hexane/22DMB (6:1 in mol)	Pressure-driven	Liquid	Liquid	25	5.60×10^{-2}	1.37
Monolayer graphene [5b]	Hexane/22DMB (152:1 in mol)	Pressure-driven	Liquid	Liquid	25	-	2.13
6FDA-DAM CMC [11]	Hexane/2MP/23DMB (1:1:1 in mol）	Osmotically-driven	Liquid	Liquid	22	1.90×10^{-7}	41.3
MB_2 [12]	Hexane/22DMB/23DMB/3MP (1:1:1:1 in mol）	Vapor separation	Vapor	Vapor	100	5.72×10^{-6}	1.64

MB_4 [12]	Hexane/22DMB/ 23DMB/3MP (1:1:1:1 in mol)	Vapor separation	Vapor	Vapor	100	5.14×10^{-6}	1.38
MB_5 [12]	Hexane/22DMB/ 23DMB/3MP (1:1:1:1 in mol)	Vapor separation	Vapor	Vapor	70	7.91×10^{-6}	1.46
MB_5 [12]	Hexane/22DMB/ 23DMB/3MP (1:1:1:1 in mol)	Vapor separation	Vapor	Vapor	100	8.47×10^{-6}	1.52
MB_5 [12]	Hexane/22DMB/ 23DMB/3MP (1:1:1:1 in mol)	Vapor separation	Vapor	Vapor	130	9.03×10^{-6}	1.53
MB_5 [12]	Hexane/22DMB/ 23DMB/3MP (1:1:1:1 in mol)	Vapor separation	Vapor	Vapor	170	9.27×10^{-6}	1.53
MB_6 [12]	Hexane/22DMB/ 23DMB/3MP (1:1:1:1 in mol)	Vapor separation	Vapor	Vapor	100	8.62×10^{-6}	1.33
MB_9	Hexane/22DMB/ 23DMB/3MP (1:1:1:1 in mol)	Vapor separation	Vapor	Vapor	100	7.38×10^{-6}	1.11

MB_15 [12]	Hexane/22DMB/ 23DMB/3MP (1:1:1:1 in mol)	Vapor separation	Vapor	Vapor	100	9.39×10^{-6}	1.15
H-ZSM-5 zeolite [13]	Hexane/22DMB (1:1)	Vapor separation	Vapor	Vapor	~127	1.00×10^{-4}	1000
H-ZSM-5 zeolite [13]	Hexane/22DMB (1:1)	Pervapovation	Liquid	Vapor	~127	3.00×10^{-3}	35
MFI-type zeolitic [14]	Hexane/23DMB (1:1)	Pervapovation	Liquid	Vapor	~30	1.50×10^{-5}	120
Al-bttotb MOF [15]	Hexane/22DMB (1:1 in mass)	Pervaporation	Liquid	Vapor	25	1.29×10^{-4}	3.85

SI Reference

[1] Y. Liang, C. Liu, M. Zhao, R. Wang, D. Zhang, C. Wang, L. Zhou, L. Wang, Z. Xie, J. Peng, L. Liu, *ACS Appl. Mater. Interfaces* **2020**, *12* (18), 20714, https://doi.org/10.1021/acsami.9b22456.

[2] A. Palma-Cando, U. Scherf, *ACS Appl. Mater. Interfaces* **2015**, *7* (21), 11127, https://doi.org/10.1021/acsami.5b02233.

[3] a) Y. Lu, L. Zhang, L. Shen, W. Liu, R. Karnik, S. Zhang, *Proc. Natl. Acad. Sci. U. S. A.* **2021**, *118* (37), e2111360118; b) Y. Lu, W. Liu, K. Wang, S. Zhang, *J. Mater. Chem. A* **2022**, *10* (37), 20101.

[4] a) B. Liang, H. Wang, X. Shi, B. Shen, X. He, Z. A. Ghazi, N. A. Khan, H. Sin, A. M. Khattak, L. Li, Z. Tang, *Nat. Chem.* **2018**, *10* (9), 961, https://doi.org/10.1038/s41557-018-0093-9; b) J. G. Wijmans, R. W. Baker, *J. Membr. Sci.* **1995**, *107* (1), 1, https://doi.org/https://doi.org/10.1016/0376-7388(95)00102-I.

[5] a) B. E. Poling, G. H. Thomson, D. G. Friend, R. L. Rowley, W. V. Wilding, *Perry's Chemical Engineers' Handbook. Section 2*, McGraw-Hill Publishing, **2008**; b) C. Cheng, S. A. Iyengar, R. Karnik, *Nature nanotechnology* **2021**, *16* (9), 989, https://doi.org/10.1038/s41565-021-00933-0.

[6] A. F. Barton, *CRC handbook of solubility parameters and other cohesion parameters*, CRC press, **1991**.

[7] a) M. Jahandar Lashaki, M. Fayaz, S. Niknaddaf, Z. Hashisho, *J. Hazard. Mater.* **2012**, *241-242*, 154, https://doi.org/https://doi.org/10.1016/j.jhazmat.2012.09.024; b) S. Karan, Z. Jiang, A. G. Livingston, *Science* **2015**, *348* (6241), 1347, https://doi.org/doi:10.1126/science.aaa5058; c) S. Van der Perre, T. Van Assche, B. Bozbiyik, J. Lannoeye, D. E. De Vos, G. V. Baron, J. F. M. Denayer, *Langmuir* **2014**, *30* (28), 8416, https://doi.org/10.1021/la501594t; d) Y. Weng, S. Qiu, L. Ma, Q. Liu, M. Ding, Q. Zhang, Q. Zhang, T. Wang, *Catalysts* **2015**, *5* (4), 2147.

[8] B. Van der Bruggen, J. Schaep, D. Wilms, C. Vandecasteele, *J. Membr. Sci.* **1999**, *156* (1), 29, https://doi.org/https://doi.org/10.1016/S0376-7388(98)00326-3.

[9] Y. Wang, S.-H. Ho, H.-W. Yen, D. Nagarajan, N.-Q. Ren, S. Li, Z. Hu, D.-J. Lee, A. Kondo, J.-S. Chang, *Biotechnol. Adv.* **2017**, *35* (8), 1049, https://doi.org/https://doi.org/10.1016/j.biotechadv.2017.06.001.

[10] C. Xue, F. Liu, M. Xu, J. Zhao, L. Chen, J. Ren, F. Bai, S.-T. Yang, *Biotechnol. Bioeng.* **2016**, *113* (1), 120, https://doi.org/https://doi.org/10.1002/bit.25666.

[11] H. Seo, S. Yoon, B. Oh, Y. G. Chung, D.-Y. Koh, *Adv. Sci.* **2021**, *8* (17), 2004999, https://doi.org/https://doi.org/10.1002/advs.202004999.

[12] P. S. Bárcia, A. Ferreira, J. Gascon, S. Aguado, J. A. C. Silva, A. E. Rodrigues, F. Kapteijn, *Microporous Mesoporous Mater.* **2010**, *128* (1), 194, https://doi.org/https://doi.org/10.1016/j.micromeso.2009.08.023.

[13] S. Sommer, T. Melin, J. L. Falconer, R. D. Noble, *J. Membr. Sci.* **2003**, *224* (1), 51, https://doi.org/https://doi.org/10.1016/j.memsci.2003.06.002.

[14] T. Matsufuji, K. Watanabe, N. Nishiyama, Y. Egashira, M. Matsukata, K. Ueyama, *Ind. Eng. Chem. Res.* **2000**, *39* (7), 2434, https://doi.org/10.1021/ie000026z.

[15] W. Yang, X. Yang, Y. Wang, R. Hou, Q. Gong, Y. Pan, *J. Membr. Sci.* **2022**, *661*, 120916, https://doi.org/https://doi.org/10.1016/j.memsci.2022.120916.
